# Nitrogen-Based Linkers with a Mesitylene Core: Synthesis and Characterization

**DOI:** 10.3390/molecules26195952

**Published:** 2021-09-30

**Authors:** Lucian Gabriel Bahrin, Alina Nicolescu, Sergiu Shova, Narcisa Laura Marangoci, Lucian Mihail Birsa, Laura Gabriela Sarbu

**Affiliations:** 1 Intelcentre, Petru Poni Institute of Macromolecular Chemistry, Romanian Academy, 41A Aleea Grigore Ghica Voda, 700487 Iasi, Romania; alina@icmpp.ro (A.N.); shova@icmpp.ro (S.S.); nmarangoci@icmpp.ro (N.L.M.); 2 Department of Chemistry, Alexandru Ioan Cuza University of Iasi, 11 Carol I Blvd., 700506 Iasi, Romania; lbirsa@uaic.ro

**Keywords:** C-C coupling, cycloaddition reaction, tetrazole, tritopic organic linker, 1,2,3-triazole

## Abstract

Mesitylene was used as a core in seven new tritopic nitrogen containing linkers. Three of the linkers, each containing three nitrile groups, were obtained through Suzuki, Sonogashira and Heck-type coupling reactions. Next, these were converted to tetrazol-5-*yl* moieties by the cycloaddition of sodium azide to the nitrile functionalities. The last linker, containing three 1,2,3-triazol-4-*yl* moieties, was synthesized by the Huisgen cycloaddition of phenyl azide to the corresponding alkyne. The latter was obtained via a Corey–Fuchs reaction sequence from the previously reported formyl derivative. As the proof of concept for their potential in MOF design, one of the nitriles was used to build an Ag-based network.

## 1. Introduction

Azoles are an important class of heterocyclic compounds that are ubiquitous in nature. In living matter/the natural world, members of this class are essential building blocks of many biomolecules, including DNA, proteins and alkaloids. Moreover, azoles often act as pharmacophores and can be found in many drugs, including antibacterials, antifungals and antihypertensives [1,2]. One important representative of this class is the tetrazole ring. Comprising four nitrogen atoms and one carbon atom, this aromatic heterocycle has found many uses in the modern world, ranging from explosives [3] to carboxylic acids bioisosters in drug design [4]. Owing to its wide range of metal binding possibilities, the tetrazole ring is also used in metal–organic frameworks (MOFs), a relatively new class of crystalline, porous materials comprising metal nodes or clusters bound together by organic linkers. The interest in MOFs is ever-increasing due to their wide range of possible applications, such as in gas storage and separation, catalysis, luminescence, water treatment and so on [5,6,7,8,9,10]. MOFs built using tetrazole-based linkers have been shown to be effective in storing carbon dioxide [11,12]. Furthermore, tetrazole-based linkers have found their way into the design of MOFs, with potential applications in dye adsorption and degradation [13,14], drug-sensing [15], heavy metal sensing [16], ethylene separation [17] and energetic materials [18,19].

Another equally important azole representative is 1,2,3-triazole. Consisting of three nitrogen atoms and two carbon atoms, this heterocycle is readily prepared via the azide alkyne Huisgen cycloaddition, a prime example of a click-reaction that is widely used in organic synthesis/bioactive molecules design as a means to bind two fragments together due to the relatively easy synthetic procedures and high yields. Similar to the tetrazole ring, the 1,2,3-triazole ring is a popular choice in MOF linker design. As such, 1,2,3-triazole-based linkers were used in MOFs with various potential applications, such as photocatalytic hydrogen generation [20], selective carbon dioxide adsorption [21], selective dye adsorption [22], water sensing [23], metal sensing [24] and enantioselective materials design [25,26].

Our continued interest in the field of azole derivatives [27,28,29,30,31], as well as MOF design [32,33,34], led to the synthesis and characterization of four new azole-based linkers with a mesitylene core.

## 2. Results and Discussion

**Scheme 1:**
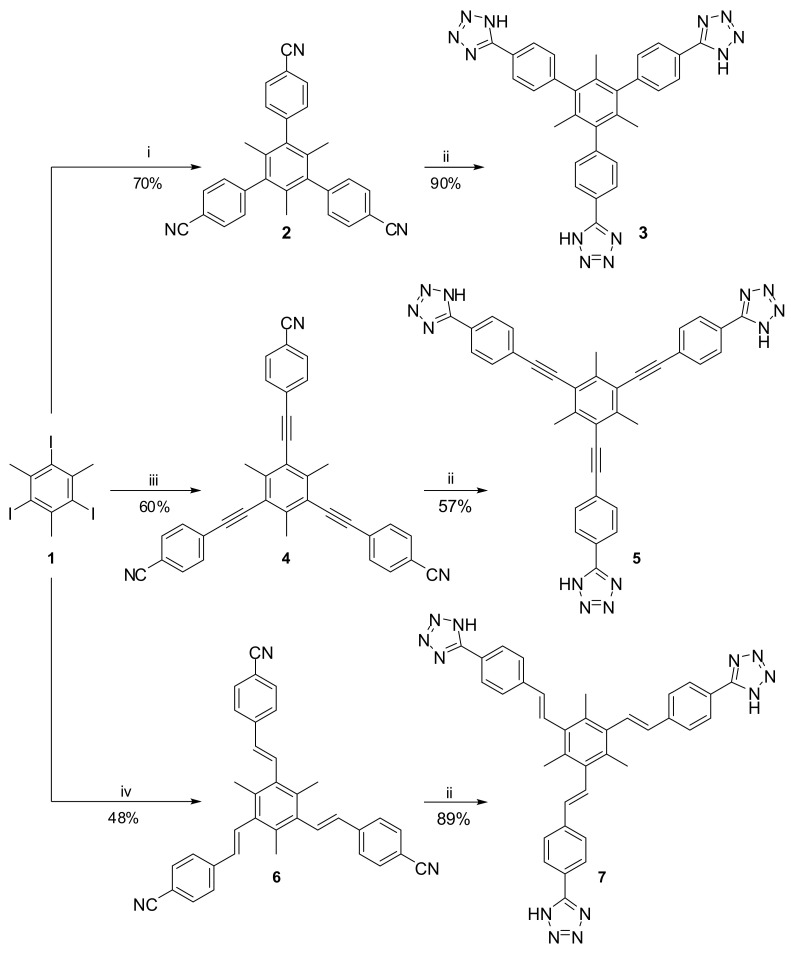
The synthesis of tetrazolyl linkers **3**, **5** and **7**. Reaction conditions are as follows: (**i**) 4-cyanophenylboronic acid, K_2_CO_3_, PhCH_3_/EtOH/H_2_O, XPhosPd G4, 100 °C, 6 h; (**ii**) NaN_3_, triethylamine hydrochloride, 120 °C, 24 h; (**iii**) 4-ethynylbenzonitrile, TEA, XPhosPd G4, 55 °C, 72 h; (**iv**) 4-cyanostyrene, K_2_CO_3_, 1,4-dioxane/H_2_O, XPhosPd G4, 110 °C, 40 h.

The first three linkers, each bearing three nitrile moieties, were obtained through a C-C coupling reaction between triiodomesitylene **1** and the corresponding coupling partner, yielding derivatives **2**, **4** and **6**, as depicted in Scheme 1. The synthesis of **2** involved a Suzuki coupling between triiodomesitylene **1** and 4-cyanophenylboronic acid. The reaction was run in a mixture of toluene, ethanol and water using potassium carbonate as a base and XPhosPd G4 as a catalyst. Tris(cyano) derivative **2** was thus obtained as a colorless solid in a 70% isolated yield by use of flash chromatography. Nitrile **4** was obtained through a Sonogashira type coupling reaction between triiodomesitylene **1** and 4-cyanophenylacetylene. Attempts to conduct the reaction in mixtures of DMF/TEA and THF/TEA led to the incomplete conversion of the starting material. Moreover, when copper was employed as a co-catalyst, the undesired Glaser-coupling reaction products were identified in the reaction mixtures. However, when the reaction was performed in neat triethylamine as both a solvent and a base without copper as a co-catalyst, nitrile **4** was obtained as a colorless solid with a 60% yield upon separation by flash chromatography. In order to obtain **6**, a Heck reaction was employed using triiodomesitylene **1** and 4-cyanostyrene as coupling partners. The procedure was performed in a mixture of refluxing dioxane and water using potassium carbonate as a base. Following workup, the desired product **6** was isolated as a colorless solid in a 57% yield.

All three nitriles were then converted to the corresponding tetrazolyl linkers **3**, **5** and **7** using sodium azide, triethylamine hydrochloride and DMF as a solvent. The reactions were performed at 120 °C over the course of 24 h, during which the color changed from light yellow to orange-brown. Upon the cooling of the reaction mixtures, the three desired linkers were obtained as brown solids after precipitation in water. In order to purify them, each was dissolved in a 10% solution of ammonium hydroxide and filtered to remove any insoluble impurities. The pH was then adjusted to 1–2 using 10% hydrochloric acid, and the precipitate was filtered and washed thoroughly with water and air-dried, yielding the purified tetrazolyl derivatives in moderate to good yields. Linker **7** was obtained as a mixture of *E* and *Z* stereoisomers.

**Scheme 2:**
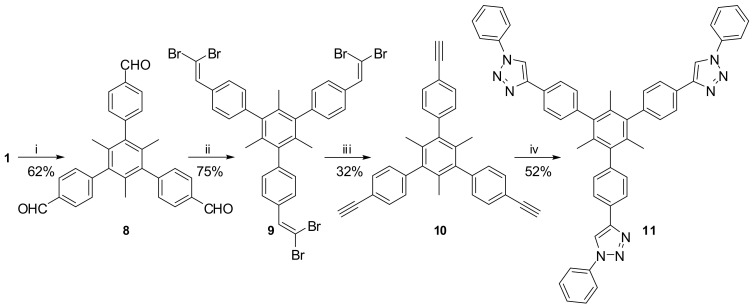
The synthesis of triazolyl linker **11**. Reaction conditions are as follows: (**i**) 4-formylphenylboronic acid, K_2_CO_3_, i-PrOH/H_2_O, XPhosPd G4, reflux, 48 h; (**ii**) CBr_4_, PPh_3_, CH_2_Cl_2_, 0 °C, 16 h; (**iii**) n-BuLi, THF, −78 °C, 1 h; (**iv**) PhN_3_, 1,4-dioxane, [Cu(phen)(PPh_3_)_2_]NO_3_, 60 °C, 24 h.

The fourth linker, bearing three 1,2,3-triazol-4-yl moieties, was obtained according to Scheme 2.

The Suzuki coupling of triiodomesityl **1** with 4-formylbenzeneboronic acid in a mixture of i-propanol and water as a solvent afforded aldehyde **8** as a colorless solid. The spectral data for **8** is in good accordance with previous reports [35]. Next, aldehyde **8** was converted to alkyne **10** through a Corey–Fuchs reaction sequence. The first step involved the synthesis of olefin **9** by the treatment of **8** with tetrabromomethane and triphenylphosphine at 0 °C. Following flash chromatography, **9** was obtained in a 75% yield as an off-white powder. When reacted with n-butyllithium at −78 °C, olefin **9** was converted to the desired alkyne **10**. Once again, after flash chromatography, **10** was isolated in a 32% yield as a colorless powder. Finally, **10** was reacted with phenyl azide in dioxane in the presence of [Cu(phen)(PPh_3_)_2_]NO_3_ as a catalyst [36] over the course of 24 h following a method previously employed by us [31]. The thick precipitate that formed was filtered and recrystallized from chloroform, yielding triazolyl linker **11** as a colorless solid in a 52% yield.

**Figure 1:**
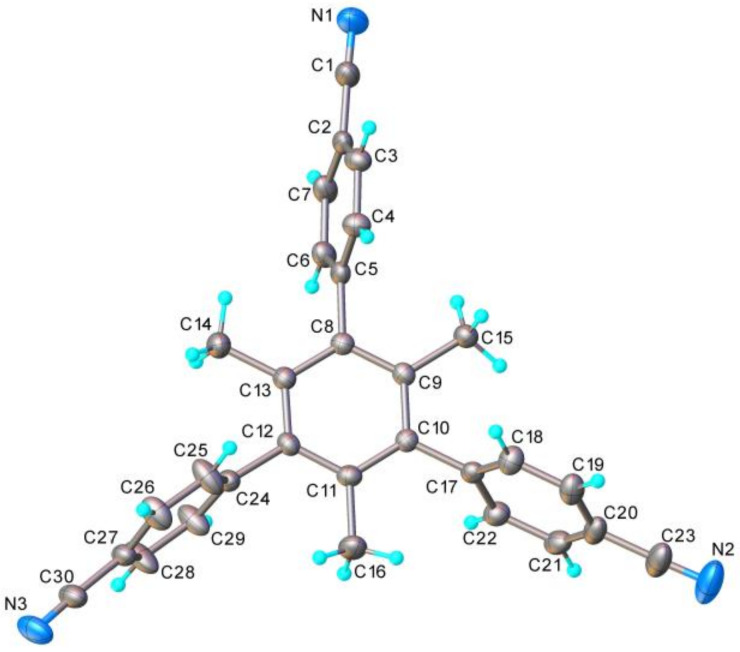
X-ray molecular structure of **2** with atom labeling and thermal ellipsoids at 40% level.

**Figure 2:**
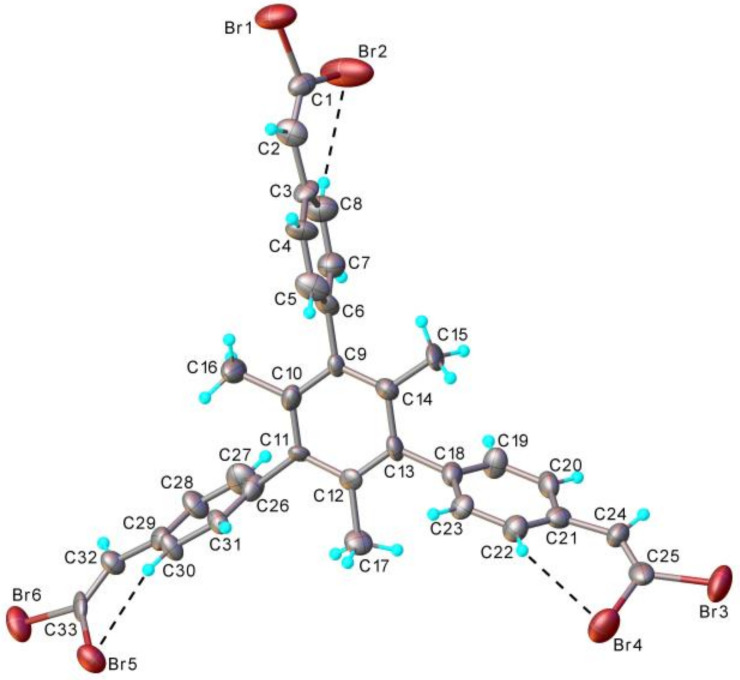
X-ray molecular structure of **9** with atom labeling and thermal ellipsoids at 40% level. H-bonds parameters: C8-H···Br2 [C8-H 0.93 Å, H···Br2 2.71 Å, C8···Br2 3.336(6) Å, ∠C8HBr2 125.3°]; C22-H···Br4 [C22-H 0.93 Å, H···Br4 2.68 Å, C22···Br4 3.337(6) Å, ∠C22HBr4 128.4°]; C30-H···Br5 [C30-H 0.93 Å, H···Br5 2.69 Å, C30···Br5 3.312(6) Å, ∠C30HBr5 124.7°].

**Figure 3:**
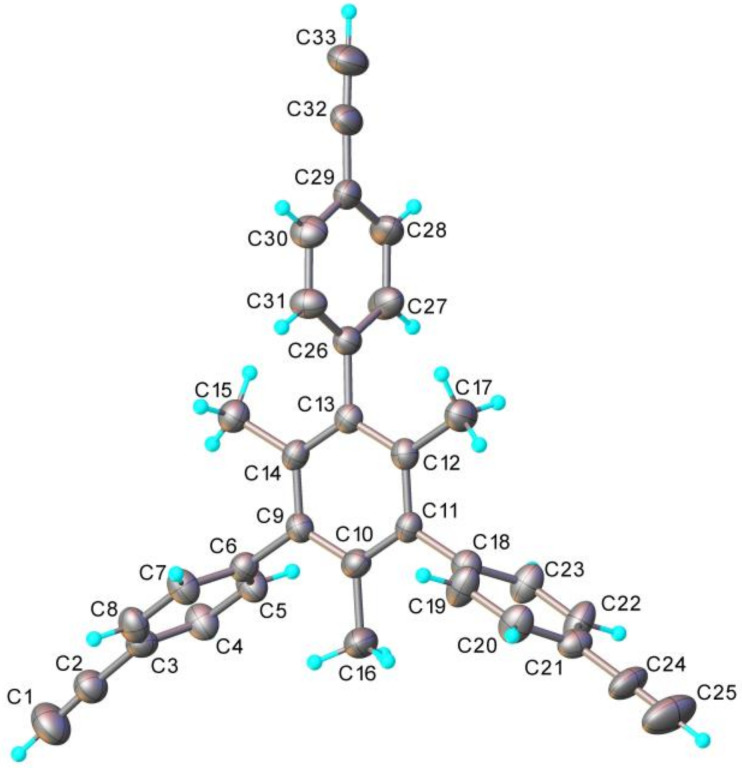
X-ray molecular structure of **10** with atom labeling and thermal ellipsoids at 40% level.

The crystal and molecular structure for compounds **2**, **9** and **10** were demonstrated by the single crystal diffraction method. The recrystallization of **2** from ethanol and layering hexane over a dichloromethane solution for **9** and **10** yielded suitable single crystals. The results of X-ray analysis for **2**, **9** and **10** are illustrated in Figure 1, Figure 2 and Figure 3, respectively. The bond distances and angles are summarized in the Supplementary Information section. According to the X-ray crystallography, the chemical composition of **2**, **9** and **10** is consistent with that proposed on the basis of the spectroscopic methods mentioned above. All three compounds exhibit a molecular crystal structure with one crystallographic unit in the asymmetric part of the unit cell. Compounds **2** and **9** crystallize with 1 and 1.5 solvate water molecules per asymmetric unit, respectively. There are no co-crystallized solvate molecules in the crystal of compound **10**. Due to steric hindrance, in each of these molecules, the three peripheral phenyl groups are almost perpendicular to the central mesitylene fragment. All three structures can be found in the Cambridge Crystallographic Data Centre using the CCDC No. 2099115 (**2**), 2099116 (**9**) and 2099117 (**10**), respectively.

**Figure 4:**
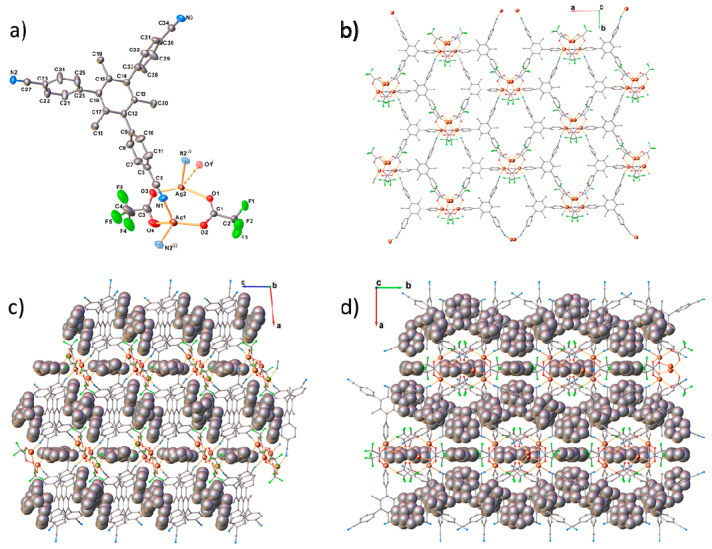
(**a**) The asymmetric unit in the crystal structure of {[Ag_2_L(Tf)_2_]·3C_7_H_8_}_n_ (**12**) with atom labelling and thermal ellipsoids at 40% level; (**b**) View of 2D coordination network; (**c**) Partial view of the crystal structure along *b* axis with space filling of guest toluene molecules; (**d**) Partial view of the crystal structure along *c* axis. H-atoms are not shown. Symmetry codes: *^i^* 1 − *x*, 1 − *y*, 1 − *z*; *^ii^* 0.5 − *x*, *y* − 0.5, 0.5 − *z*; *^iii^* 0.5 + *x*, 0.5 + *y*, *z*.

As the proof of concept that the new derivatives can be employed in the design of metal–organic frameworks, we decided to use nitrile **2** as a linker in a silver-based network. For this, a solution of silver trifluoroacetate in toluene was carefully laid over a solution of **2** in chloroform. We expected MOF crystals to form at the interface of the two solutions; however, after 72 h at room temperature, this was not the case. We, therefore, decided to allow some of the solvent mixture to slowly evaporate, and, for this, the cap of the vial was unscrewed halfway so that it would not form an air-tight seal anymore. After several weeks, we found that colorless crystals had begun to form on the bottom of the vial. The result of X-ray diffraction study for compound **12** is shown in Figure 4. The asymmetric part of the unit cell (Figure 4a) comprises two Ag^+^ cations, two acetate anions, one tris(cyano) derivative **2** and three molecules of toluene that are disordered within several positions. The tris(cyano) derivative is coordinated as a tridentate ligand and fulfills the function of bridging linker between binuclear {Ag_2_(Tf)_2_} coordination nodes (Tf-trifluoroacetate anion), consolidated via two carboxylate groups acting in a μ_2_-*k*^2^*O*:*O*′ mode. The coordination environment of the argentum atoms is slightly different. Ag1 exhibits a distorted tetrahedral coordination provided by two nitrogen atoms (Ag1-N1 2.338(4) Å, Ag1-N3(0.5 + *x*, 0.5 + *y*, *z*) 2.274(4) Å) and two carboxylate oxygen atoms (Ag1-O2 2.239(3), Ag1-O4 2.289(4) Å). The coordination environment of the Ag2 atom involves two carboxylate oxygen atoms (Ag2-O1 2.281(3), Ag2-O3 2.219(3) Å) and one nitrigen atom (Ag2-N2 2.343(4) Å) in a trigonal planar geometry and weekly coordinated oxygen atom O1 from the adjacent unit at Ag1-O1(1 − *x*, 1 − *y*, 1 − *z*) = 2.68797(5) Å distance. Thus, it could be assumed that the coordination of Ag2 is characterized as 3 + 1 in a distorted tetrahedral geometry. The analysis of the crystal packing shows the prsesence of two-dimensional coordination polymers (Figure 4b) arranged parallel to 110 plane. It is also noteworthy that, due to the above mentioned Ag1-O1′ contact, the 2D polymers are further extended to form a 3D coordination network. The partial view of the crystal packing showing the topology of the 3D network is illustrated in Figure 4c,d. As can be seen, the compound {[Ag_2_L(Tf)_2_]}_n_ (**12**) represets a neutral, potentially porous framework with crossing channels running along *b* and *c* axis (Figure 4b,c, respectively). As calculated with the map tool available in Olex2 program using the probe of 1.2 Å radius and 0.2 Å, the volume of empty channels constitutes 45% of the unit cell volume. The structure of **12** can be accessed at the Cambridge Crystallographic Data Centre using the CCDC No. 2106774.

## 3. Materials and Methods

### 3.1. Chemistry

The NMR spectra have been recorded on a Bruker NEO 400 instrument (Bruker BioSpin, Rheinstetten, Germany) operating at 400.1 and 100.6 MHz for ^1^H and ^13^C nuclei using either a 5 mm multinuclear inverse detection z-gradient probe or a 5 mm four nuclei direct detection z-gradient probe (mainly for 1D ^13^C spectra). Chemical shifts are reported in δ units (ppm) and were referenced to the internal deuterated solvent (DMSO-*d*_6_ reference at 2.51 ppm (^1^H) and 39.4 (^13^C) and CDCl_3_ reference at 7.26 ppm (^1^H) and 77.0 ppm (^13^C)). The signals were assigned based on 2D NMR homo- and heteronuclear correlations, such as H,H-COSY, H,C-HSQC and H,C-HMBC, recorded using standard pulse sequences in the version with z-gradients, as delivered by Bruker with TopSpin 4.0 PL8 spectrometer control and processing software. IR spectra were recorded on a Shimadzu IRTracer-100 instrument (Shimadzu U.S.A. Manufacturing, Inc., Canby, OR, USA). Mass spectra were recorded on a Thermo Scientific ISQ LT instrument (Thermo Fisher Scientific Inc., Waltham, MA, USA). Elemental analyses (C, H) were conducted using a CE440 Elemental Analyzer (Exeter Analytical, Coventry, United Kingdom); the results were found to be in good agreement (±0.30%) with the calculated values. Melting points were measured on a KSPI melting-point meter and are uncorrected (A.KRÜSS Optronic, Hamburg, Germany).

#### 3.1.1. Synthesis of **2**

In a round-bottom flask, potassium carbonate (1.65 g, 12 mmol) was dissolved in water (10 mL). To this, ethanol (10 mL) and toluene (20 mL) were added, and the resulting mixture was degassed by bubbling nitrogen for 15 min. After degassing, triiodomesitylene (0.5 g, 1 mmol) was added, followed by 4-cyanophenylboronic acid (0.57 g, 3.9 mmol), XPhosPdG4 (25 mg, 0.03 mmol, 3 mol%) and XPhos (14 mg, 0.03 mmol, 3 mol%). The mixture thus obtained was stirred at 100 °C for 6 h, during which the color of the reaction changed from yellow to black. It was then allowed to cool down to room temperature, and water (20 mL) was added to it. The crude product was extracted with dichloromethane (3 × 30 mL), and the organic phase was dried over sodium sulfate and the solvent was removed under reduced pressure. The remaining residue was separated using flash chromatography, with dichloromethane as an eluent, yielding the desired product **2** as a colorless solid (300 mg, 70% isolated yield). M.p. 237–238 °C. ^1^H NMR (400 MHz, CDCl_3_): δ= 7.75 (6H, d, ^3^*J* = 8.2 Hz, H-2), 7.32 (6H, d, ^3^*J* = 8.2 Hz, H-3), 1.64 (9H, s, CH_3_) ppm. ^13^C NMR (100 MHz, CDCl_3_): 146.3 (C-4), 138.6 (C-5), 132.9 (C-6), 132.6 (CH-2), 130.2 (CH-3), 118.7 (CN), 111.2 (C-1), 19.4 (CH_3_) ppm. FT-IR (ATR): ῦ = 2228, 1605, 1504, 1396, 1267, 1107, 1020, 957, 847, 561 cm^−1^. EI-MS *m*/*z* (%): 423 (M^+^, 100), 408 (40), 305 (15), 291 (10).

#### 3.1.2. Synthesis of **4**

Triethylamine (20 mL) was added to a Schlenk tube and degassed by bubbling nitrogen for 15 min. Triiodomesitylene (0.5 g, 1 mmol), 4-ethynylbenzonitrile (0.46 g, 3.6 mmol) and XPhosPdG4 (50 mg, 0.06 mmol, 6 mol%) were then added, and the flask was capped with a silicone septum and heated at 55 °C under a nitrogen atmosphere for 72 h. The triethylamine was then removed under reduced pressure, and the remaining residue was separated using flash chromatography, with a mixture of dichloromethane-hexane as an eluent, yielding **4** as a colorless solid (300 mg, 60% isolated yield). M.p. 298–299 °C. ^1^H NMR (400 MHz, CDCl_3_): δ= 7.67 (6H, d, ^3^*J* = 8.5 Hz, H-2), 7.64 (6H, d, ^3^*J* = 8.5 Hz, H-3), 2.74 (9H, s, CH_3_) ppm. ^13^C NMR (100 MHz, CDCl_3_): 143.4 (C-8), 132.2 (CH-2), 131.9 (CH-3), 128.1 (C-4), 120.9 (C-7), 118.4 (CN), 111.8 (C-1), 96.1 (C-5), 90.6 (C-6), 20.4 (CH_3_) ppm. FT-IR (ATR): ῦ = 2230, 2201, 1601, 1501, 1406, 1261, 1179, 1103, 1015, 822, 550, 444 cm^−1^. EI-MS *m*/*z* (%): 495 (M^+^, 40), 420 (100), 390 (30), 241 (40).

#### 3.1.3. Synthesis of **6**

In a round-bottom flask, a solution of potassium carbonate (0.84 g, 6 mmol) in water (5 mL) was prepared. 1,4-Dioxane (10 mL) was then added, and the resulting mixture was degassed by bubbling nitrogen for 15 min. Triiodomesitylene (0.5 g, 1 mmol), 4-cyanostyrene (0.47 g, 3.6 mmol), XPhosPdG4 (25 mg, 0.03 mmol, 3 mol%) and XPhos (14 mg, 0.03 mmol, 3 mol%) were then added, and the resulting reaction mixture was heated to 110 °C under a nitrogen atmosphere for 40 h. The solvent was then removed under reduced pressure, and the crude mixture was separated using flash chromatography, with a mixture of dichloromethane-hexane as an eluent, to yield **6** as a colorless solid (240 mg, 48% isolated yield). M.p. 209–210 °C. ^1^H NMR (400 MHz, CDCl_3_): δ= 7.67 (6H, d, ^3^*J* = 8.4 Hz, H-2), 7.59 (6H, d, ^3^*J* = 8.4 Hz, H-3), 7.28 (3H, d, ^3^*J* = 16.6 Hz, H-6,), 6.55 (3H, d, ^3^*J* = 16.6 Hz, H-5), 2.34 (9H, s, CH_3_) ppm. ^13^C NMR (100 MHz, CDCl_3_): 141.6 (C-4), 135.3 (C-7), 133.5 (C-8), 133.1 (CH-5), 132.5 (CH-2), 131.5 (CH-6), 126.7 (CH-3), 118.9 (CN), 110.9 (C-1), 19.1 (CH_3_) ppm. FT-IR (ATR): ῦ = 2224, 1634, 1601, 1506, 1412, 1175, 978, 808, 544, 432 cm^−1^. EI-MS *m*/*z* (%): 501 (M^+^, 50), 486 (25), 385(100), 355 (50), 254 (45), 140 (40).

#### 3.1.4. Synthesis of **3**

Nitrile **2** (0.21 g, 0.5 mmol), sodium azide (0.29 g, 4.5 mmol), triethylamine hydrochloride (0.62 g, 4.5 mmol) and DMF (10 mL) were added in a Schlenk tube. The reaction vessel was then closed with a rubber septum and connected to a flow of nitrogen through the side tube. The reaction was heated to 120 °C for 24 h, during which the color changed to brown. After 24 h, the reaction mixture was poured into a 10% solution of hydrochloric acid in water (200 mL) and stirred for 30 min. The precipitate thus formed was then filtered and dissolved into a solution of 5% sodium hydroxide in water (100 mL). The dark orange solution was then filtered, and its pH was adjusted to 1–2 using hydrochloric acid. After stirring for another 30 min, the precipitate that formed was filtered, washed thoroughly with water and air dried, yielding **3** as a brown solid (0.20 g, 90% isolated yield). M.p. > 320 °C. ^1^H NMR (400 MHz, DMSO-*d*_6_): δ= 16.92 (3H, bs, NH), 8.16 (6H, d, ^3^*J* = 8.2 Hz, H-2), 7.51 (6H, d, ^3^*J* = 8.2 Hz, H-3), 1.72 (9H, s, CH_3_) ppm. ^13^C NMR (100 MHz, DMSO-*d*_6_): 155.1 (C-7), 144.1 (C-4), 138.7 (C-5), 132.3 (C-6), 130.3 (CH-2), 127.4 (CH-3), 122.7 (C-1), 19.2 (CH_3_) ppm. FT-IR (ATR): ῦ = 3314, 2980, 1612, 1558, 1495, 1429, 1155, 1067, 1034, 999, 961, 847, 750, 542, 463 cm^−1^. EI-MS *m*/*z* (%): 552 (M^+^, 10), 457 (20), 423 (50), 316 (55), 237 (80), 223 (100).

#### 3.1.5. Synthesis of **5**

This compound was synthesized using the same procedure as for **3**, using nitrile **4** as starting material. Brown solid (0.18 g, 57% isolated yield). M.p. > 320 °C. ^1^H NMR (400 MHz, DMSO-*d*_6_, 60 °C): δ= 8.11 (6H, d, ^3^*J* = 8.4 Hz, H-2), 7.80 (6H, d, ^3^*J* = 8.4 Hz, H-3), 2.78 (9H, s, CH_3_) ppm. ^13^C NMR (100 MHz, DMSO-*d*_6_, 60 °C): 155.4 (C-9), 141.9 (C-8), 131.7 (CH-3), 126.9 (CH-2), 124.5 (C-1 and C-4), 120.5 (C-7), 96.8 (C-5), 87.9 (C-6), 19.8 (CH_3_) ppm. FT-IR (ATR): ῦ = 3314, 2980, 1612, 1558, 1495, 1429, 1155, 1067, 1034, 999, 961, 847, 750, 542, 463 cm^−1^. EI-MS *m*/*z* (%): 624 (M^+^, 5), 584 (10), 472 (10), 457 (20), 345 (15), 241 (100).

#### 3.1.6. Synthesis of **7**

This compound was synthesized using the same procedure as for **3**, using nitrile **6** as starting material. Brown solid (0.28 g, 89% isolated yield). M.p. > 320 °C. ^1^H NMR (400 MHz, DMSO-*d*_6_): δ= 16.80 (3H, bs, NH), 8.08 (6H, d, ^3^*J* = 8.2 Hz, H-2), 7.87 (6H, d, ^3^*J* = 8.2 Hz, H-3), 7.50 (3H, d, ^3^*J* = 16.6 Hz, H-6), 6.67 (3H, d, ^3^*J* = 16.6 Hz, H-5), 2.37 (9H, s, CH_3_) ppm. ^13^C NMR (100 MHz, DMSO-*d*_6_): 155.4 (C-9), 139.8 (C-4), 135.3 (C-7), 133.0 (CH-5), 132.5 (C-8), 130.0 (CH-6), 127.3 (CH-2), 127.0 (CH-3), 122.9 (C-1), 18.8 (CH3) ppm. FT-IR (ATR): ῦ = 3410, 1609, 1566, 1497, 1435, 1070, 972, 814, 745, 698, 521 cm^−1^. EI-MS *m*/*z* (%): 630 (M^+^, 4), 600 (12), 584 (15), 457 (60), 442 (15), 315 (40), 165 (100).

#### 3.1.7. Synthesis of **9**

A solution of triphenylphosphine (1.38 g, 5.28 mmol) in dry dichloromethane (10 mL) was cooled down to 0 °C and added to a solution of tetrabromomethane (0.66 g, 2.64 mmol) in dry dichloromethane (5 mL) at the same temperature under a nitrogen atmosphere. After stirring for 5 min, a solution of **8** (0.19 g, 0.44 mmol) in dry dichloromethane (3 mL) was added dropwise while maintaining 0 °C. The reaction mixture was then left to react overnight at room temperature. The next day, the solvent was removed under reduced pressure, and the crude mixture was separated using flash chromatography, with a mixture of dichloromethane/hexane as an eluent, yielding **9** as a colorless solid (0.29 g, 73%). M.p. 206–207 °C. ^1^H NMR (400 MHz, CDCl_3_): δ= 7.64 (6H, d, ^3^*J* = 8.2 Hz, H-2), 7.52 (3H, s, H-7), 7.22 (6H, d, ^3^*J* = 8.2 Hz, H-3), 1.71 (9H, s, CH_3_) ppm. ^13^C NMR (100 MHz, CDCl_3_): 142.4 (C-4), 139.3 (C-5), 136.6 (CH-7), 133.6 (C-1), 133.1 (C-6), 129.5 (CH-3), 128.7 (CH-2), 89.1 (C-8), 19.4 (CH_3_) ppm. FT-IR (ATR): ῦ = 1605, 1504, 1398, 1260, 1111, 1016, 949, 870, 822, 712, 530, 444 cm^−1^. EI-MS *m*/*z* (%): 894 (M^+^ for C_33_H_24_^79^Br_6_, 35), 449 (10), 289 (30), 200 (40), 193 (100).

#### 3.1.8. Synthesis of **10**

A solution of **9** (0.27 g, 0.3 mmol) in THF (10 mL) was cooled down to −78 °C. A solution of *n*-BuLi in hexane (1.44 mL, 3.6 mmol) was then slowly added, and the resulting mixture was stirred at −78 °C for one hour, allowed to warm up to room temperature and stirred for another hour. Aqueous hydrochloric acid (1 mL, 5%) was then added, and the reaction was stirred for 15 min. Finally, water (30 mL) was added, and the mixture was extracted with dichloromethane (3 × 20 mL), dried over anhydrous magnesium sulfate and concentrated to dryness. The crude mixture was separated using flash chromatography, with a mixture of dichloromethane/hexane as an eluent, yielding **10** as a colorless solid (0.04 g, 32%). M.p. 234–235 °C. ^1^H NMR (400 MHz, CDCl_3_): δ = 7.56 (6H, d, ^3^*J* = 8.2 Hz, H-2), 7.17 (6H, d, ^3^*J* = 8.2 Hz, H-3), 3.09 (3H, s, CH-8), 1.68 (9H, s, CH_3_) ppm. ^13^C NMR (100 MHz, CDCl_3_): 142.5 (C-4), 139.2 (C-5), 133.0 (C-6), 132.4 (CH-2), 129.4 (CH-3), 120.4 (C-1), 83.6 (C-7), 77.1(CH-8), 19.4 (CH_3_) ppm. FT-IR (ATR): ῦ = 3277, 2980, 2106, 1605, 1499, 1101, 1018, 959, 837, 660, 600, 552, 449 cm^−1^. EI-MS *m*/*z* (%): 420 (M^+^, 100), 390 (20), 289 (15), 193 (15).

#### 3.1.9. Synthesis of **11**

Phenyl azide (180 mg, 1.5 mmol) was added to a solution of acetylene **10** (105 mg, 0.25 mmol) in 1,4-dioxane (5 mL). To the resulting mixture, [Cu(phen)(PPh_3_)_2_]NO_3_ (13 mg, 0.015 mmol) was added, and the reaction medium was heated to 60 °C for 24 h. After cooling the mixture down to room temperature, the precipitate that formed was filtered off and recrystallized from chloroform (50 mL), yielding **11** as a colorless solid (102 mg, 52%). M.p. 183–184 °C. ^1^H NMR (400 MHz, DMSO-*d*_6_, 60 °C): δ= 9.24 (3H, s, H-8), 8.07 (6H, d, ^3^*J* = 8.2 Hz, H-2), 7.97 (6H, d, ^3^*J* = 7.6 Hz, H-10), 7.65 (6H, t, ^3^*J* = 7.6 Hz, H-11), 7.53 (3H, t, ^3^*J* = 7.4 Hz, H-12), 7.39 (6H, d, ^3^*J* = 8.2 Hz, H-3), 1.80 (9H, s, CH_3_) ppm. ^13^C NMR (100 MHz, DMSO-*d*_6_, 60 °C): 146.9 (C-7), 141.1 (C-4), 138.8 (C-5), 136.5 (C-9), 131.9 (C-6), 129.5 (CH-3 and CH-11), 128.5 (C-1), 128.3 (CH-12), 125.4 (CH-2), 119.8 (CH-10), 119.2 (CH-8), 18.8 (CH_3_) ppm. FT-IR (ATR): ῦ = 1670, 1597, 1504, 1489, 1225, 1040, 993, 827, 754, 687, 552 cm^−1^. EI-MS *m*/*z* (%): 777 (M^+^, 3), 725 (10), 599 (5), 472 (20), 457 (80), 330 (50), 315 (100).

#### 3.1.10. Synthesis of **12**

In a 6 mL vial with a screw cap, a solution of AgOOCCF_3_ (33 mg, 0.15 mmol) in toluene (3 mL) was carefully layered over a solution of **2** (21 mg, 0.05 mmol) in chloroform (3 mL). The cap was then screwed in place, and the vial was kept in the dark at room temperature. After 72 h, the cap was unscrewed halfway so as to allow the solvent to slowly evaporate. After several weeks in the dark, a volume of approximately 1 mL of solvent was left in the vial. At this point, the colorless crystals that had formed were filtered and air dried, yielding 45 mg of **12** (78%, based on the quantity of **2**). FT-IR (ATR): ῦ = 2247, 1651, 1605, 1431, 1186, 1136, 837, 723, 563, 465 cm^−1^.

### 3.2. X-ray Structure Determination

X-ray diffraction measurements were carried out with a Rigaku Oxford-Diffraction XCALIBUR E CCD diffractometer equipped with graphite-monochromated MoKα radiation (Oxford Diffraction Limited, Abingdon, UK). The crystals were placed at 40 mm from the detector, and 276, 238 and 219 frames were measured, each of 20 s, 20 s and 60 s, over 1° scan for **2**, **9** and **10**, respectively. The unit cell determination and data integration were carried out using the CrysAlis package of Oxford Diffraction (Rigaku Europe SE, Frankfurt, Germany) [37]. The structures were solved by Intrinsic Phasing using Olex2 [38] software (OlexSys Ltd, Durham, Great Britain) with the SHELXT [39] structure solution program and refined by full-matrix least-squares on *F*^2^ with SHELXL-2015 (University of Göttingen, Göttingen, Germany) [40] using an anisotropic model for non-hydrogen atoms. All H atoms attached to carbon were introduced in idealized positions (d_CH_ = 0.96 Å) using the riding model with their isotropic displacement parameters fixed at 120% of their riding atom. The positions of H atoms for NH groups were determined from Fourier synthesis maps and verified through the hydrogen bonds parameters. The positional parameters of the solvent toluene molecules were refined using disordered model with the help of PART, DFIX and SADI tools of SHELXL-2015 program, and necessary restraints were imposed on geometry and displacement parameters of disordered molecules. Table S1 provides a summary of the crystallographic data together with refinement details for the compounds.

## 4. Conclusions

To conclude, seven new tritopic nitrogen containing linkers with a mesitylene core were synthesized and characterized by NMR, EI-MS and IR spectroscopy. Three of them, containing nitrile functionalities, were obtained through Suzuki, Sonogashira and Heck-type C-C coupling reactions. The cyclization of the nitrile groups with sodium azide led to the formation of three new tetrazolyl–mesitylene derivatives. The last linker, containing 1,2,3-triazole units, was obtained via a Huisgen cycloaddition of phenyl azide to the corresponding alkyne. The structures of three of the new mesitylene derivatives were confirmed by single crystal X-ray diffraction. The proof of concept for the ability of the new linkers to form metal–organic frameworks was provided through the synthesis of a new MOF using silver trifluoroacetate as a metal source and nitrile **2** as an organic linker.

## Data Availability

Supplementary crystallographic data can be obtained free of charge via www.ccdc.cam.ac.uk/conts/retrieving.html (accessed on 27 September 2021) (or from the Cambridge Crystallographic Data Centre, 12 Union Road, Cambridge CB2 1EZ, UK; Fax: (+44) 1223-336-033; or deposit@ccdc.ca.ac.uk).

## Supplementary Materials

The following are available online, Figures S1–S18: 1H and 13C NMR spectra of derivatives **2**–**7** and **9**–**11**, Figures S19–S27: EI-MS spectra for derivatives **2**–**7** and **9**–**11**, Table S1: Crystal data and details of data collection, Tables S2–S9: Bond distances and angles for structures **2**, **9**, **10** and **12**, CIF files for structures **2**, **9**, **10** and **12**.

## Sample Availability

Samples of the compounds are not available from the authors.
