# Supplementary material for: Nitrogen-Based Linkers with a Mesitylene Core: Synthesis and Characterization
Source: Molecules. 2021 Sep 30;26(19):5952. doi: 10.3390/molecules26195952 (PMC8512016; doi:10.3390/molecules26195952)
Supplement: Supplementary file 1 [file molecules-26-05952-s001.zip › molecules-1390503-supplementary.pdf]

Supporting Information

for

Azole-linkers with a mesitylene core: synthesis and characterization

Lucian G. Bahrin, Alina Nicolescu, Sergiu Shova, Narcisa L. Marangoci, Lucian M. Birsa and Laura G. Sarbu

Synthetic procedures, NMR spectra and crystallographic data

Contents

NMR spectra……………………………………. S3

EI-MS spectra………………………………….. S22

X-Ray data……………………………………… S32

NMR Spectra

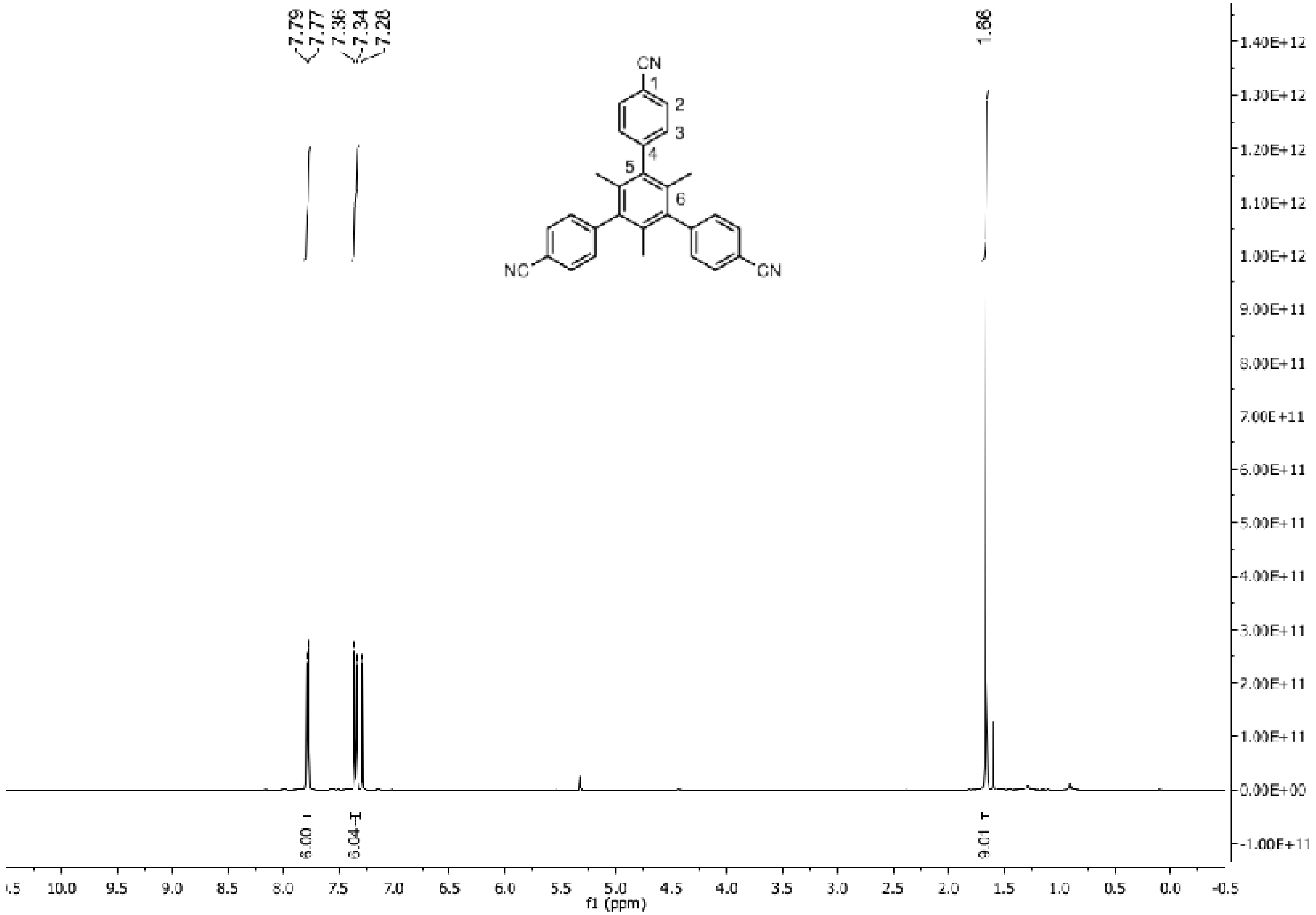

Figure S1 – The ^{1}H NMR spectrum of **2**, measured in $CDCl_3$.

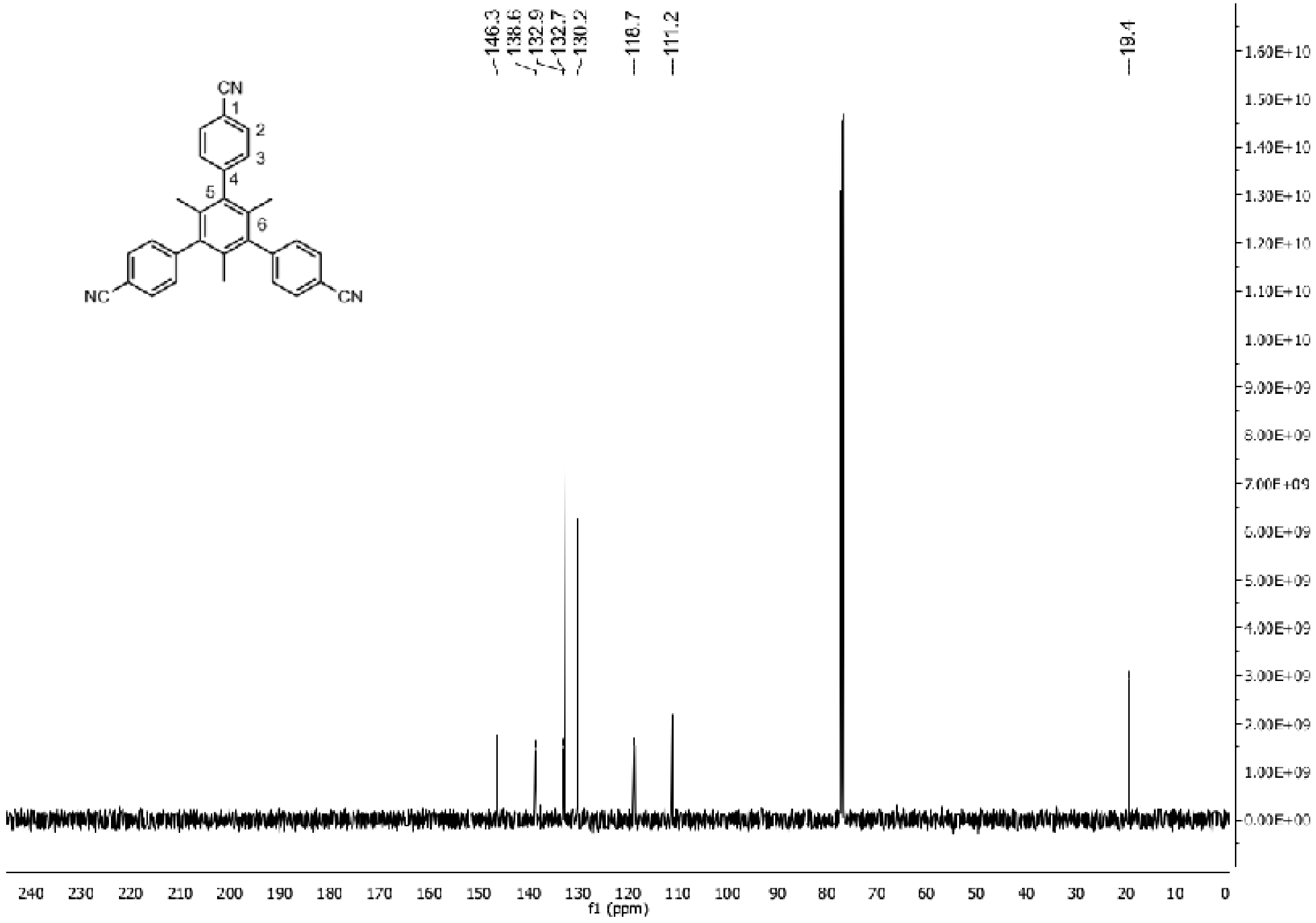

Figure S2 – The ^{13}C NMR spectrum of **2**, measured in $CDCl_3$.

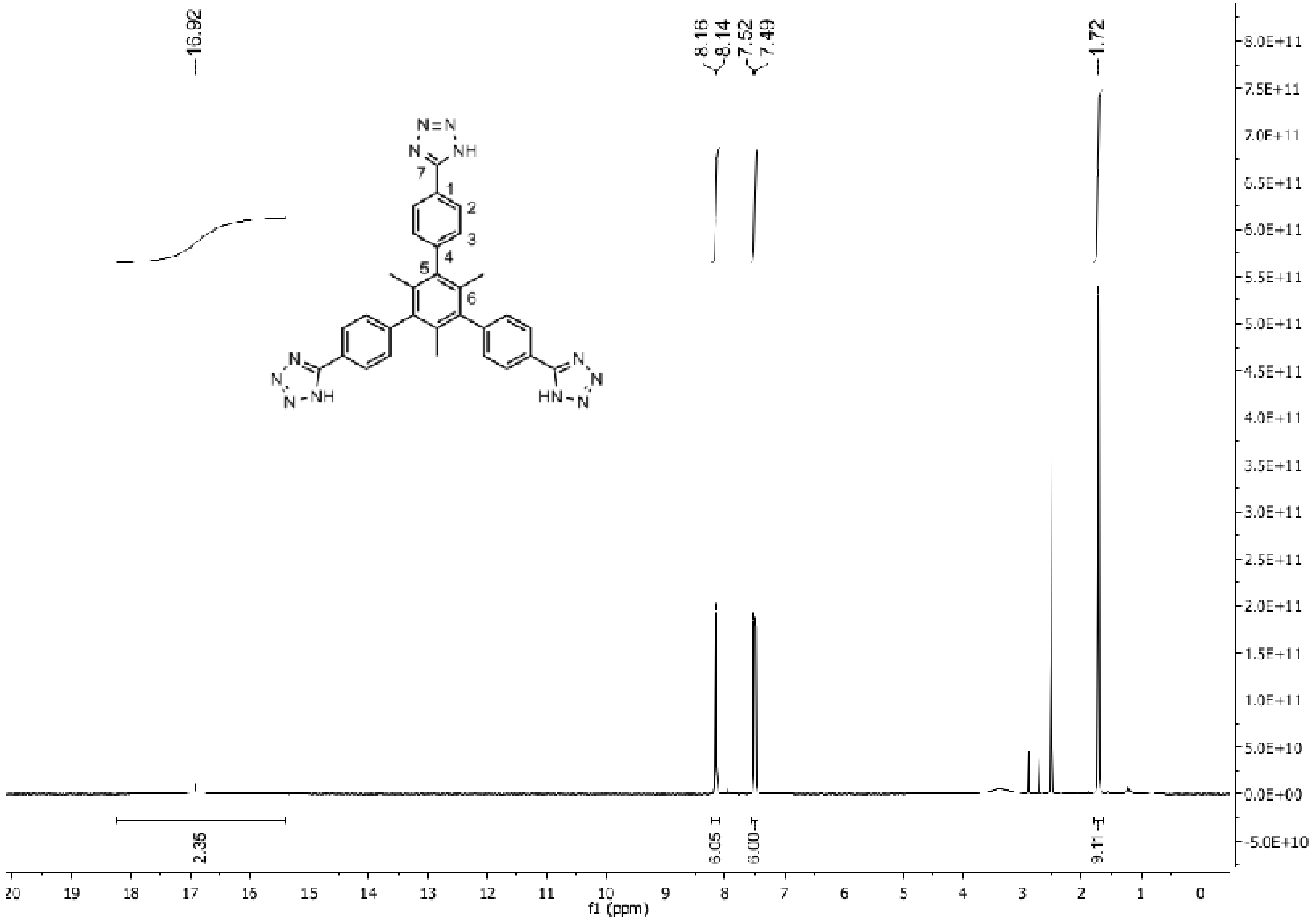

Figure S3 – The ^{1}H NMR spectrum of **3**, measured in DMSO-*d6*.

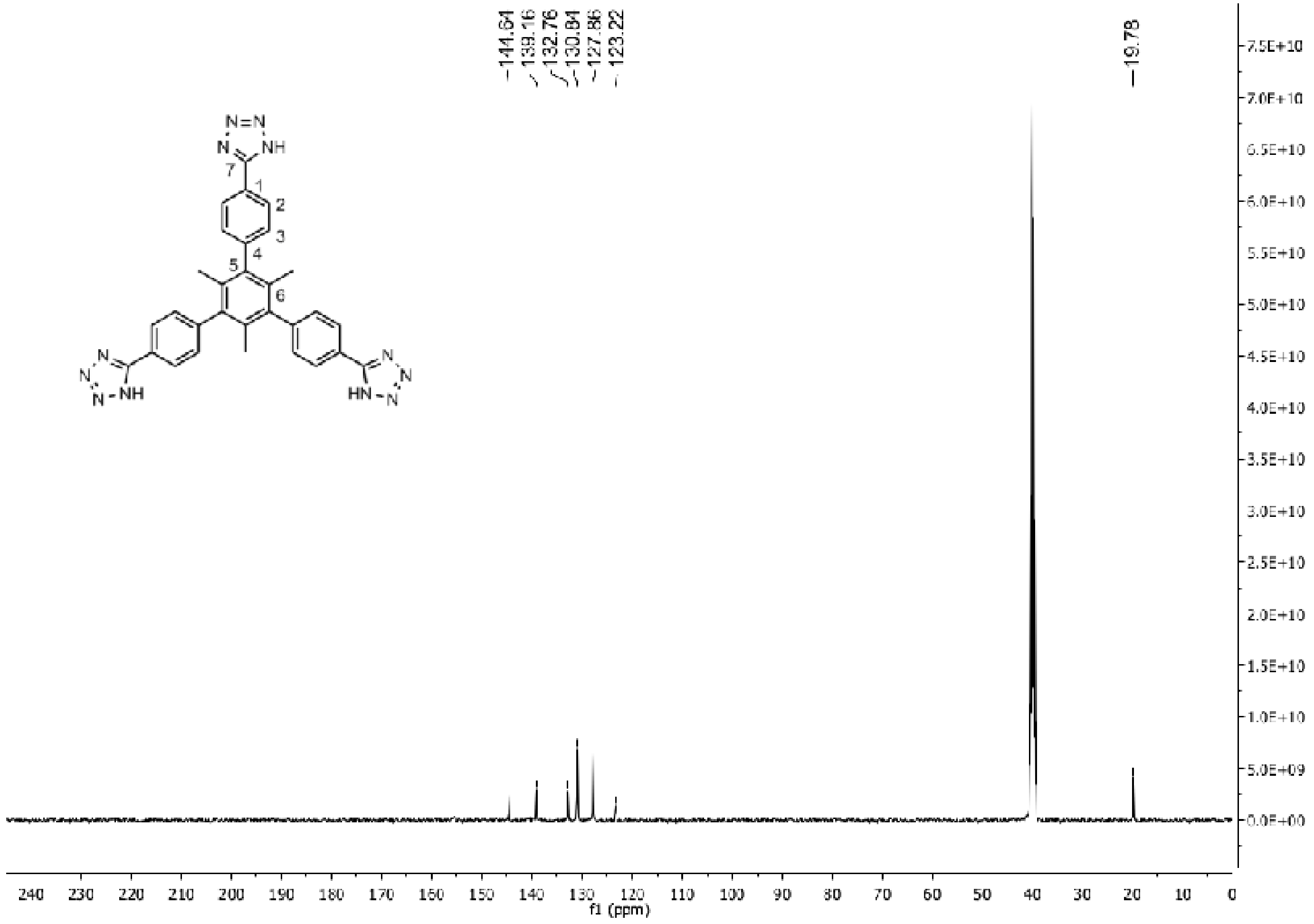

Figure S4 – The ^{13}C NMR spectrum of **3**, measured in DMSO-*d6*.

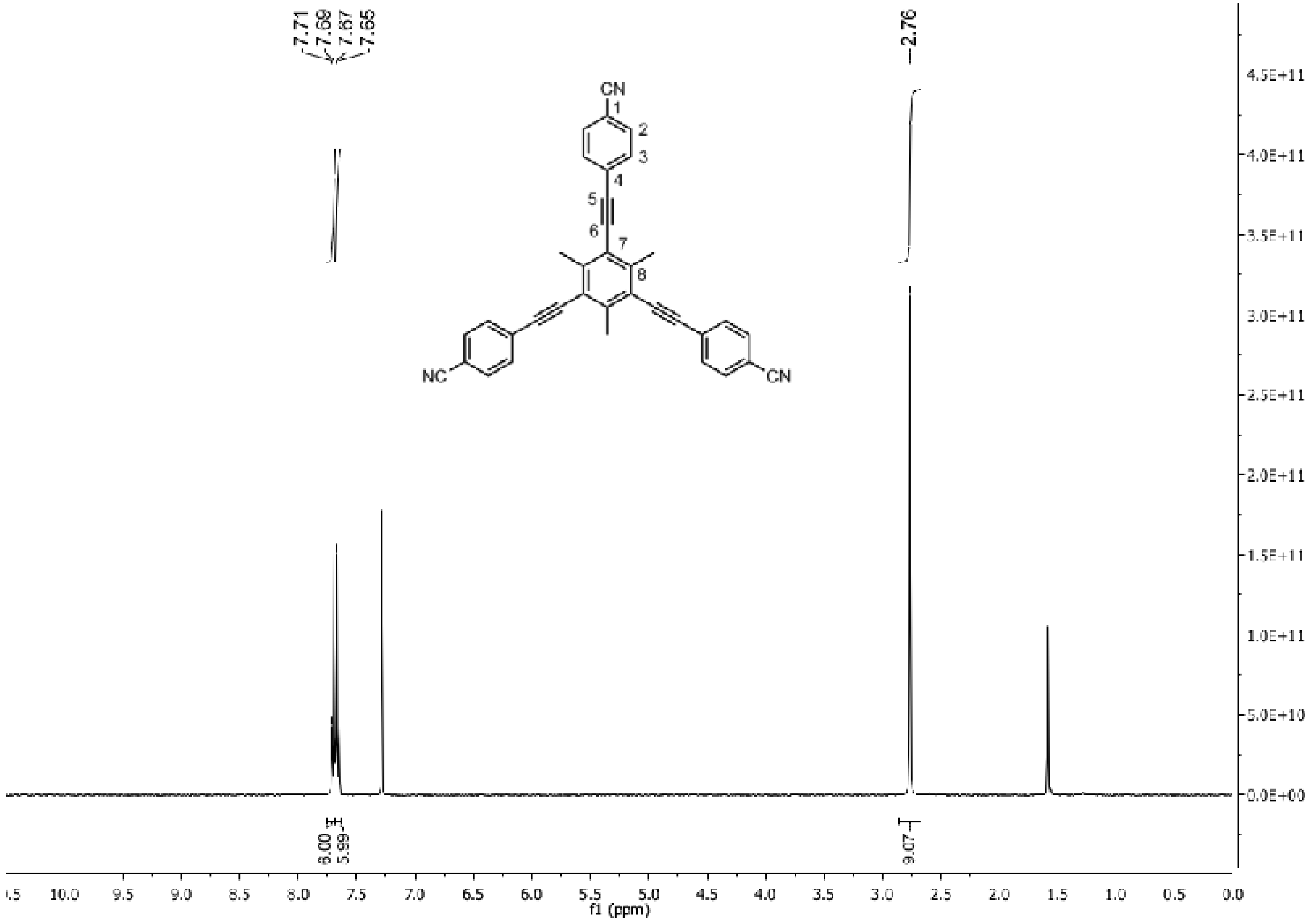

Figure S5 – The ^{1}H NMR spectrum of **4**, measured in $CDCl_3$.

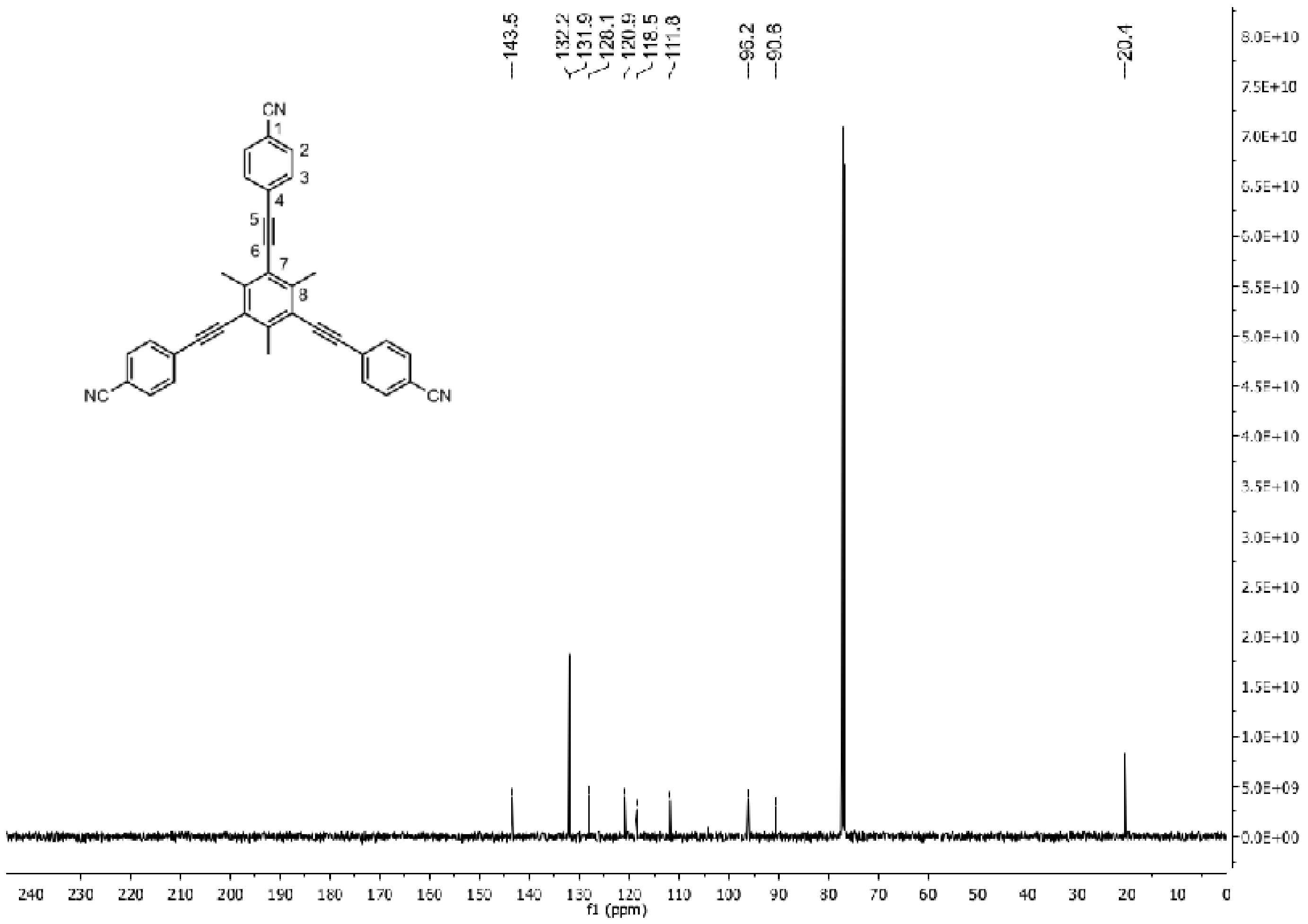

Figure S6 – The ^{13}C NMR spectrum of **4**, measured in $CDCl_3$.

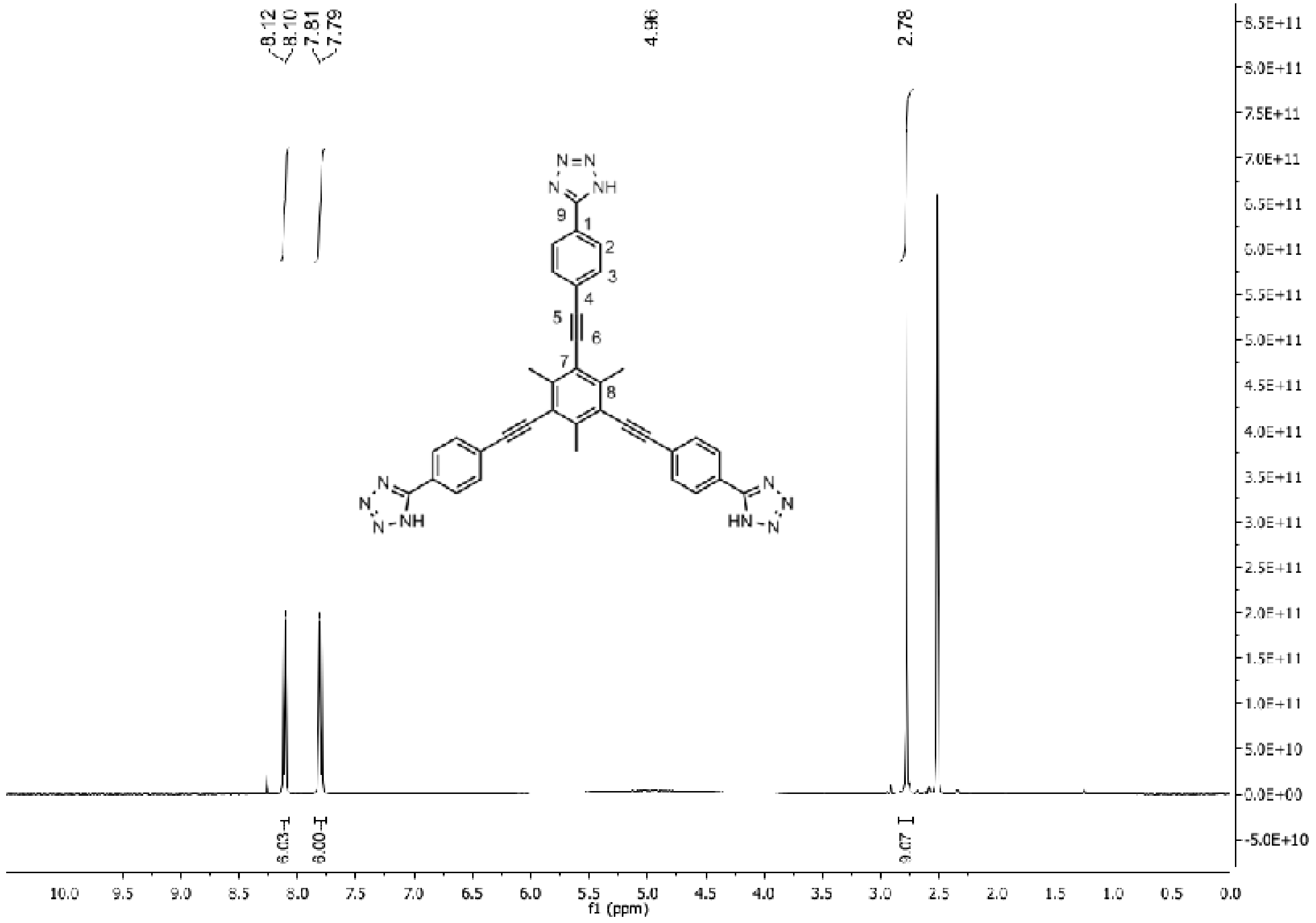

Figure S7 – The ^{1}H NMR spectrum of **5**, measured in DMSO-*d6*.

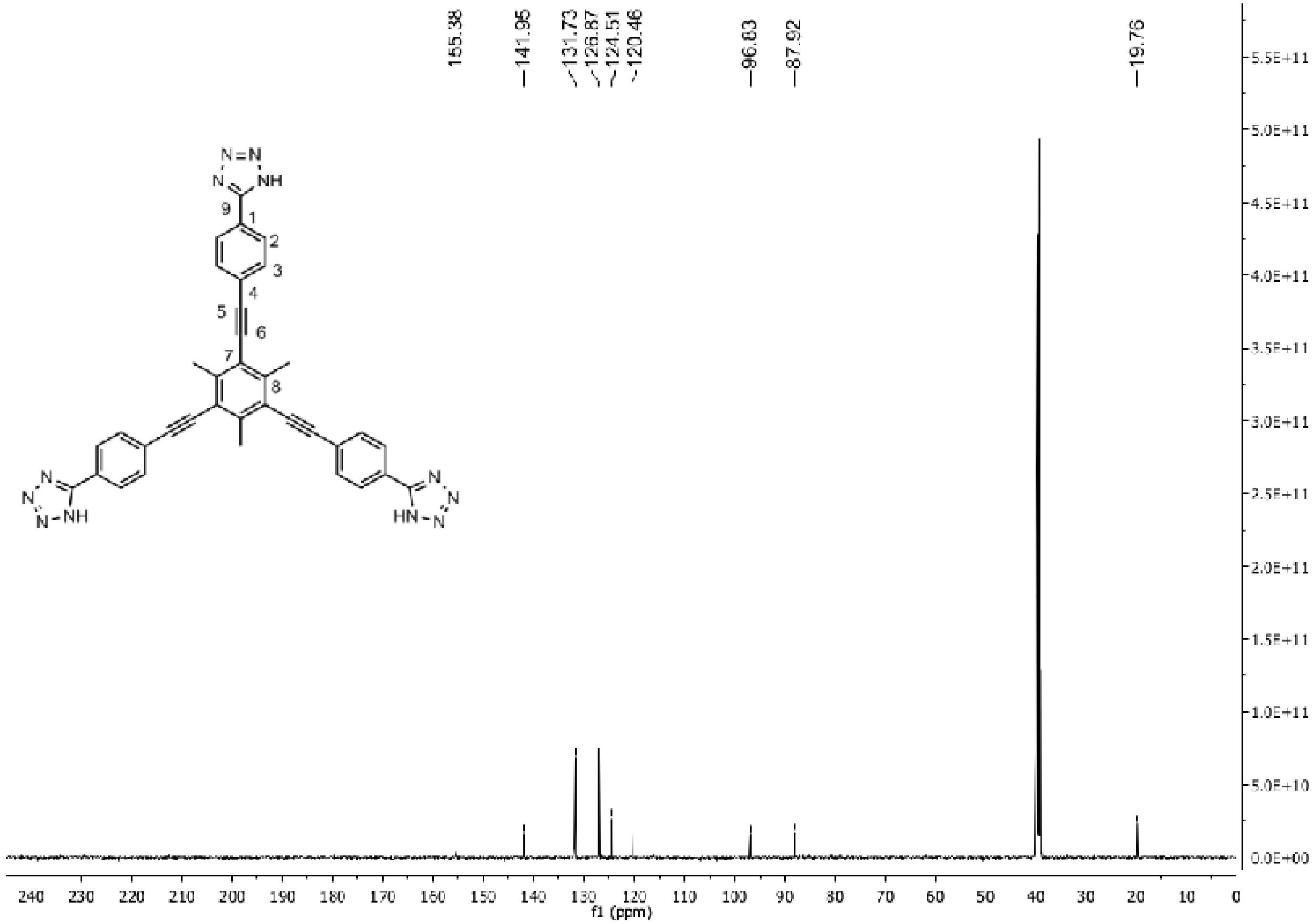

Figure S8 – The ^{13}C NMR spectrum of **5**, measured in DMSO-*d6*.

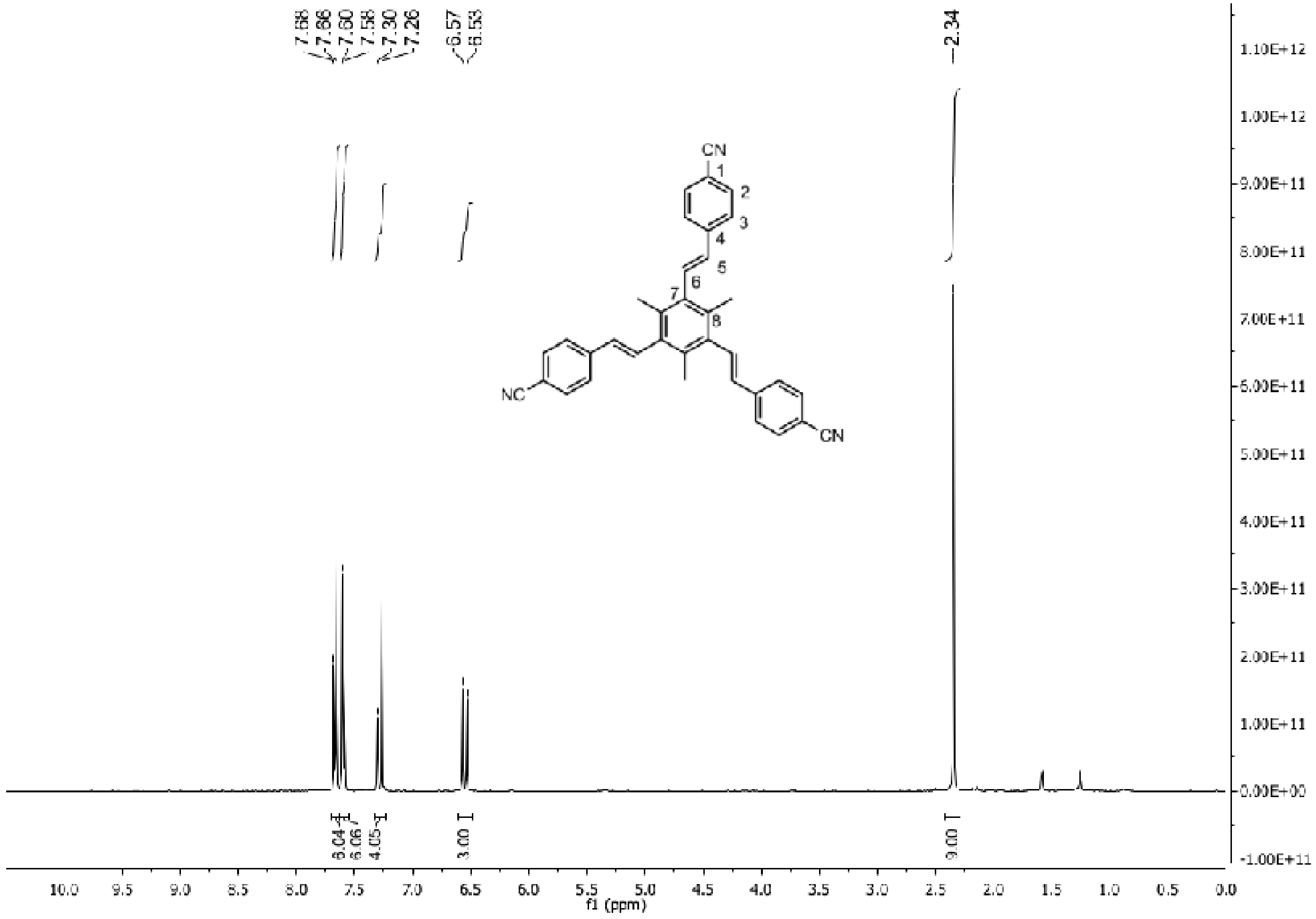

Figure S9 – The ^{1}H NMR spectrum of **6**, measured in $CDCl_3$.

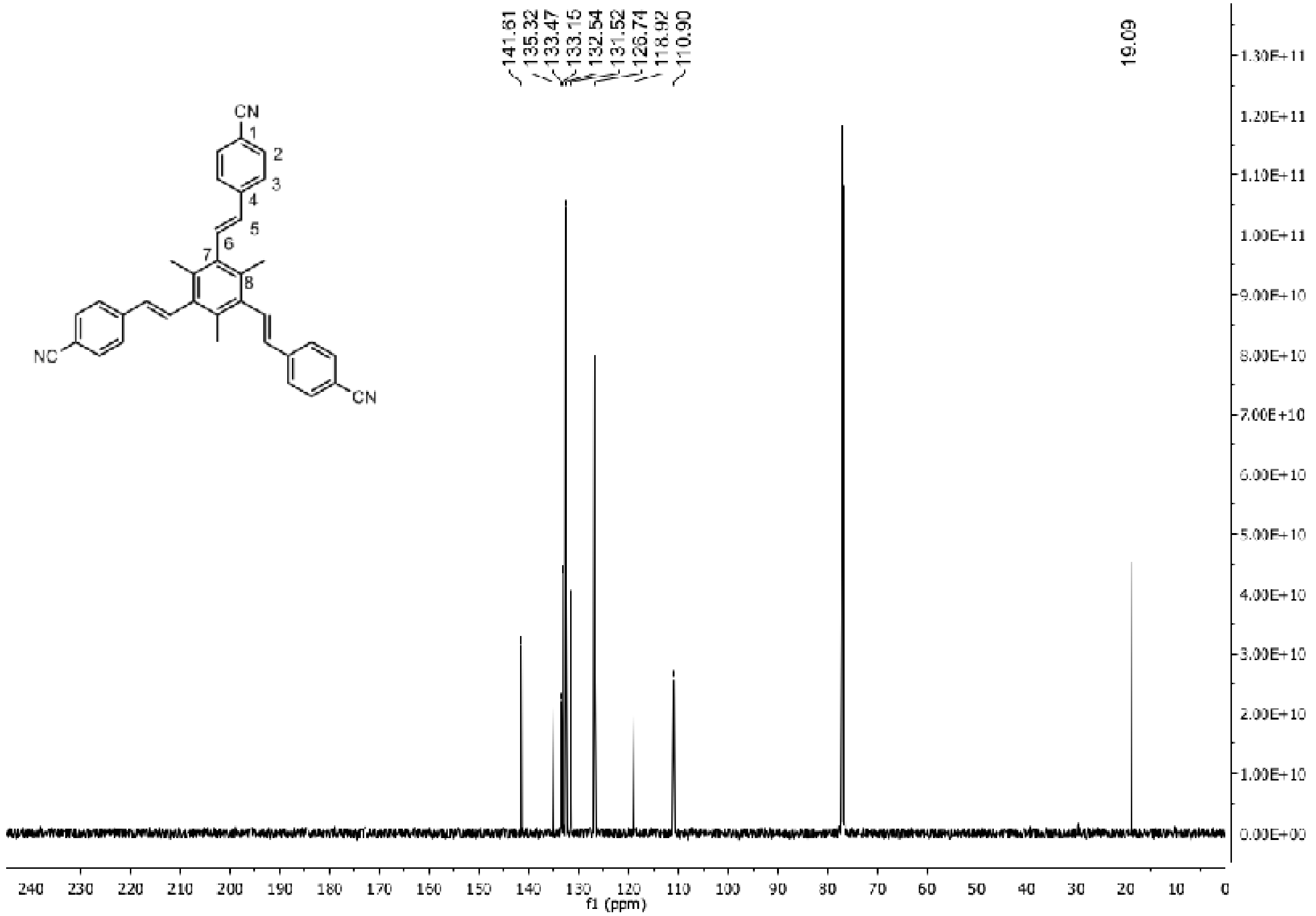

Figure S10 – The ^{13}C NMR spectrum of **6**, measured in $CDCl_3$.

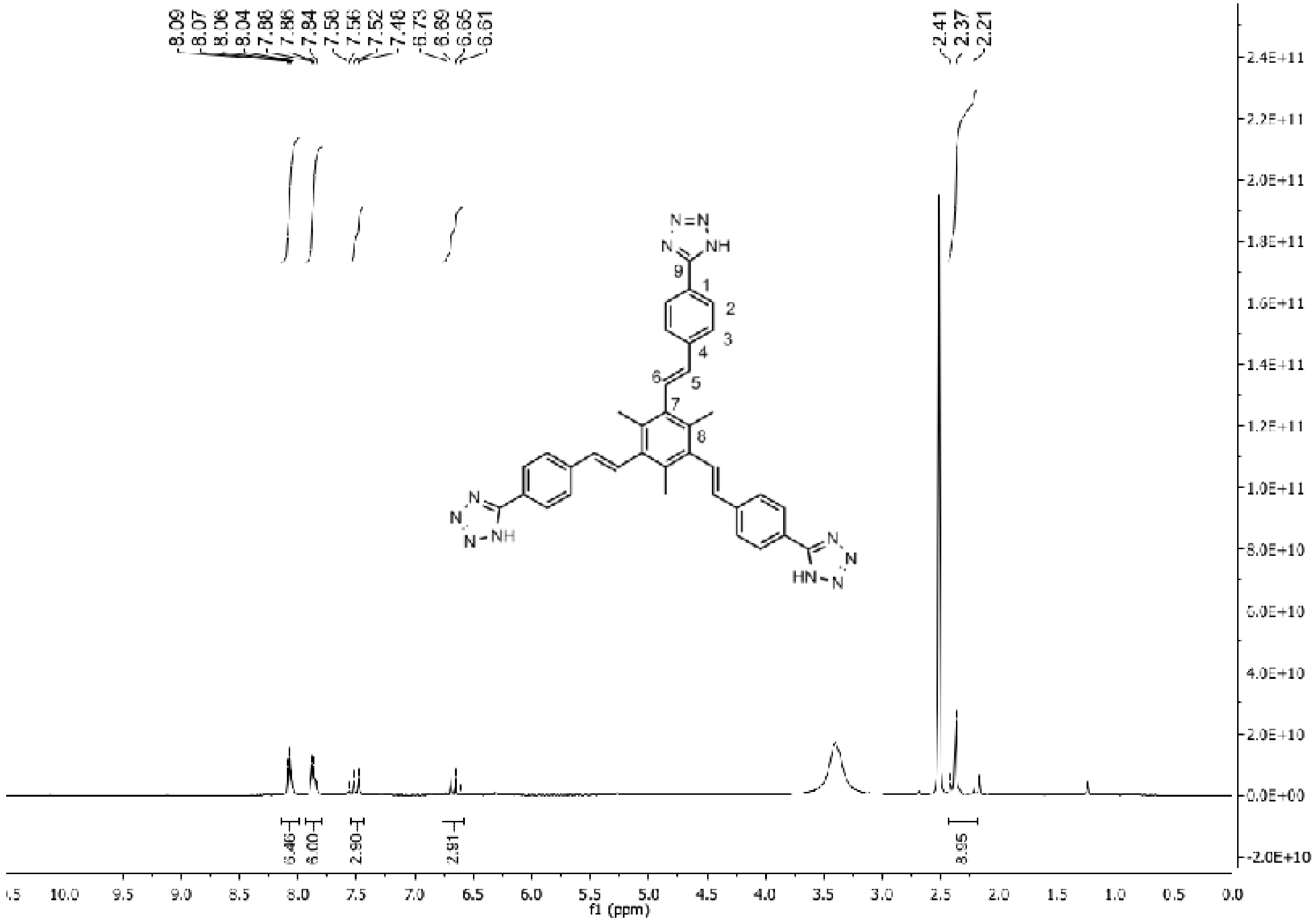

Figure S11 – The ^{1}H NMR spectrum of **7**, measured in DMSO-*d6*.

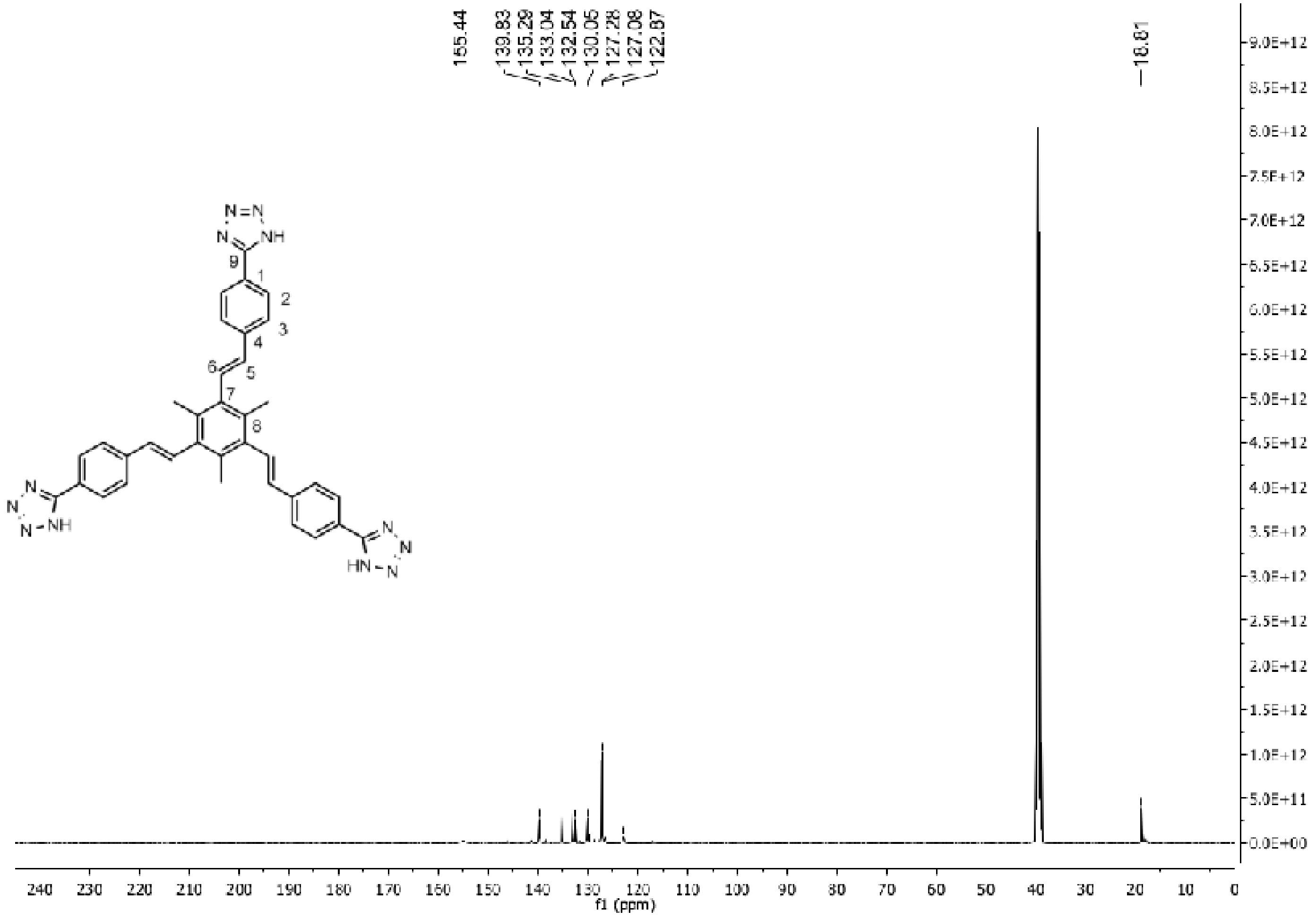

Figure S12 – The ^{13}C NMR spectrum of **7**, measured in DMSO-*d6*.

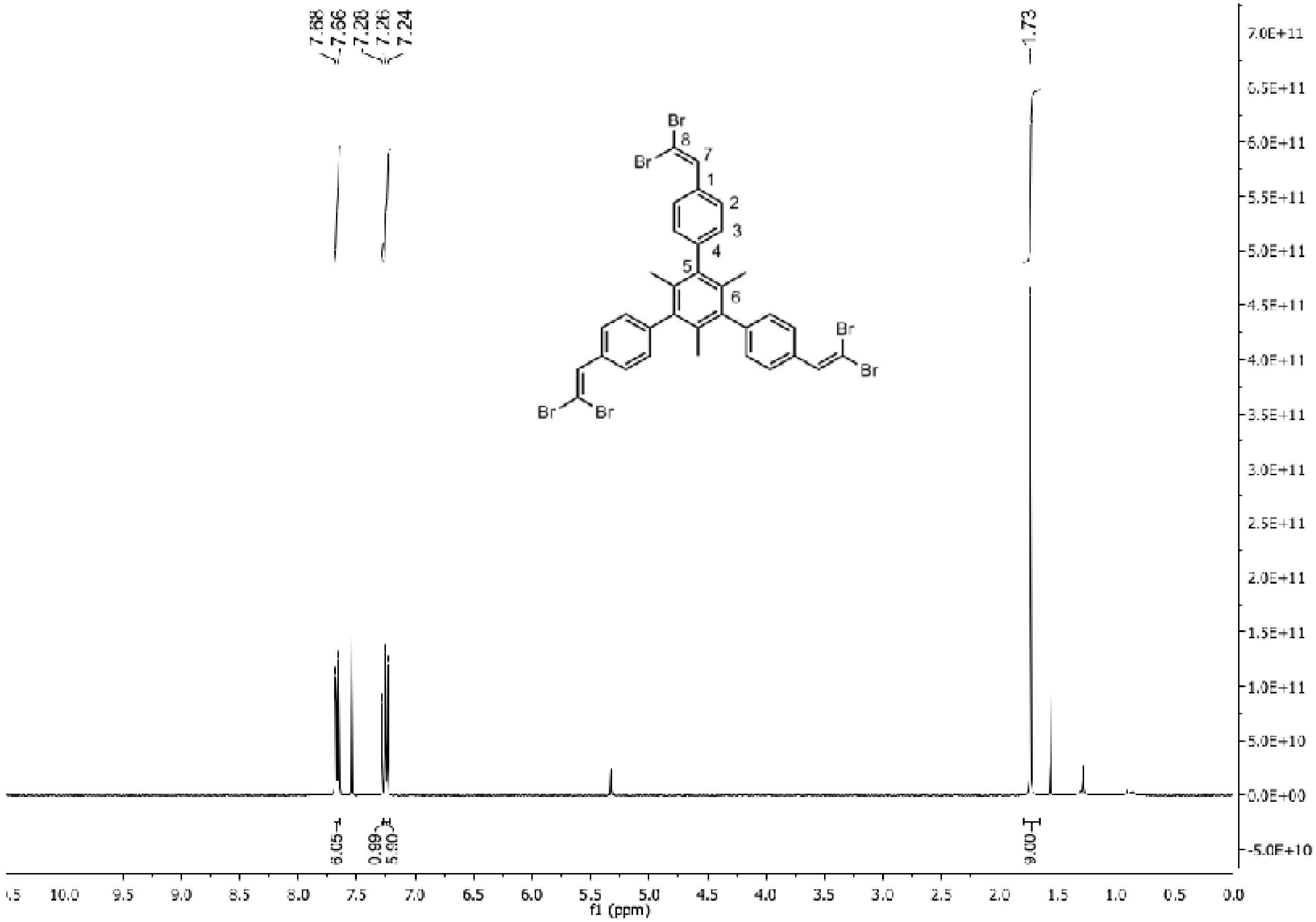

Figure S13 – The ^{1}H NMR spectrum of **9**, measured in $CDCl_3$.

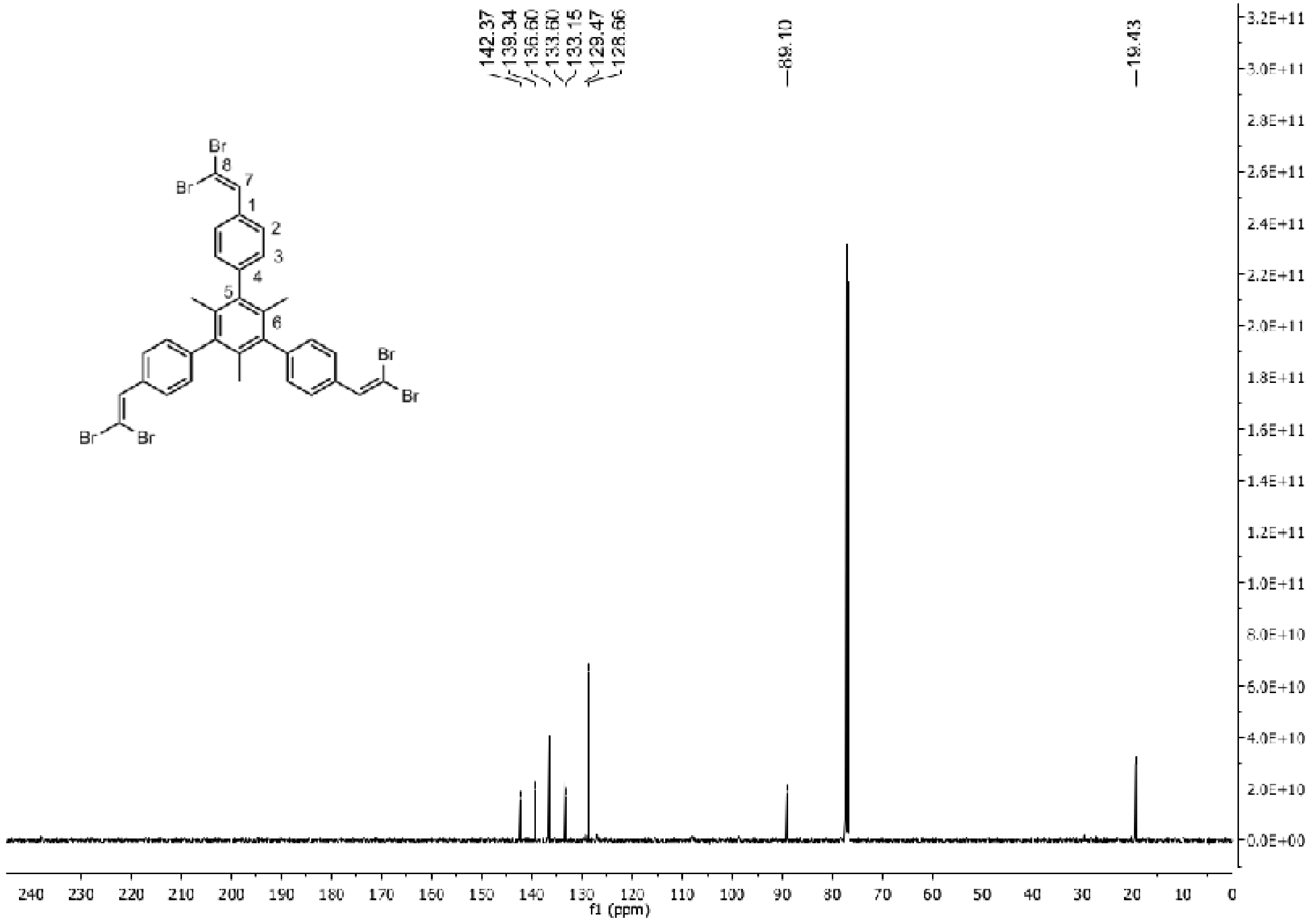

Figure S14 – The ^{13}C NMR spectrum of **9**, measured in $CDCl_3$.

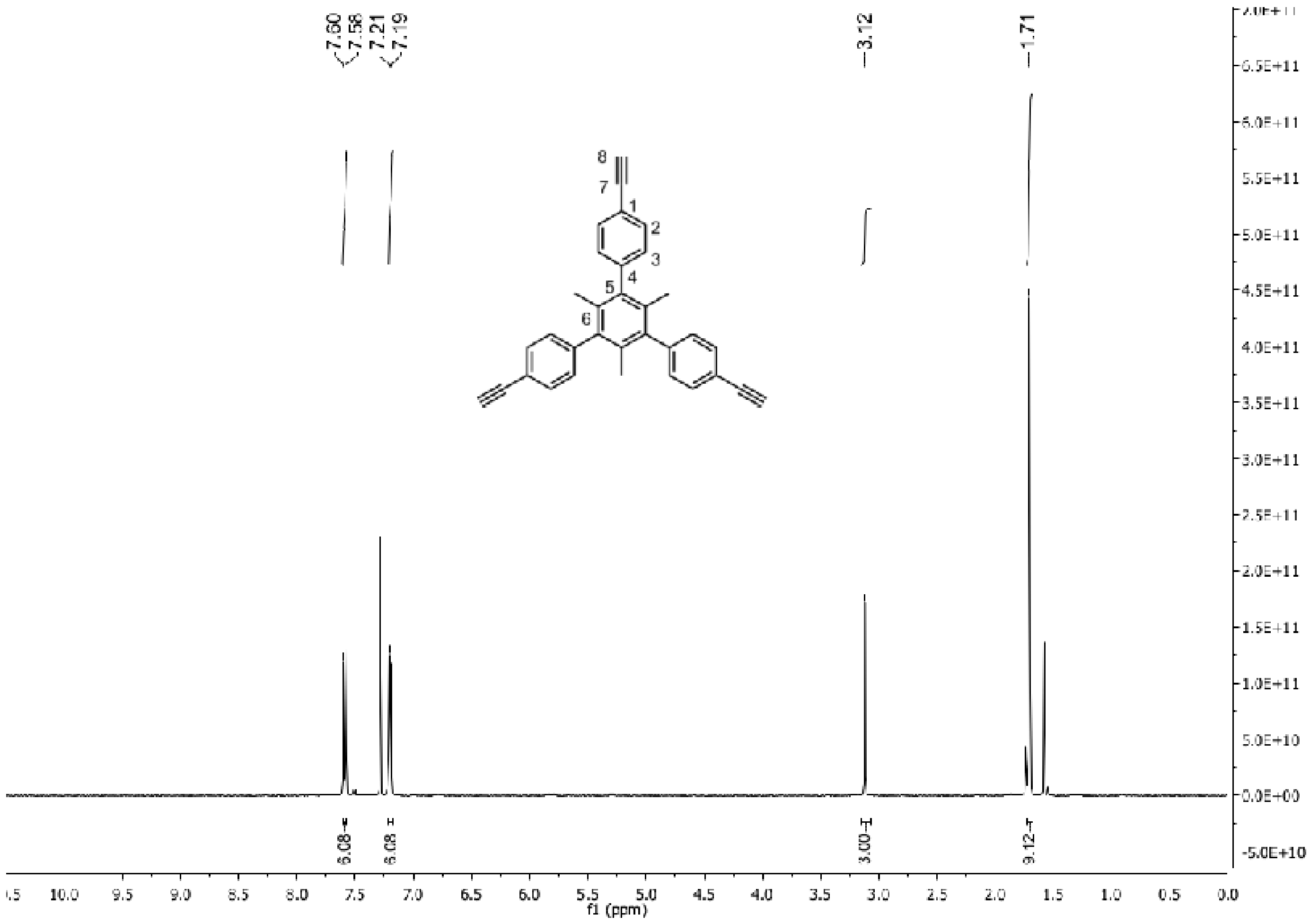

Figure S15 – The ^{1}H NMR spectrum of **10**, measured in $CDCl_3$.

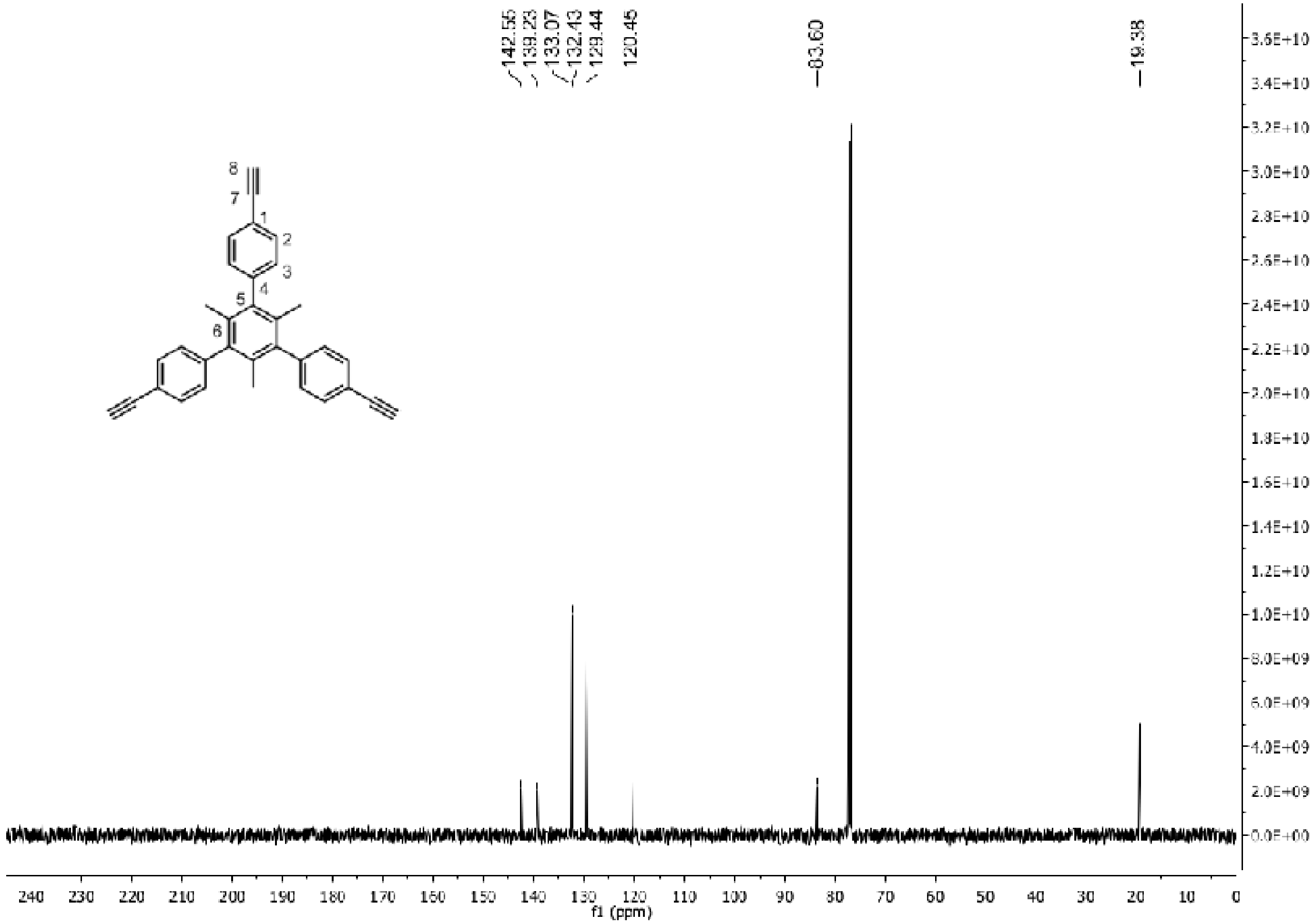

Figure S16 – The ^{13}C NMR spectrum of **10**, measured in $CDCl_3$.

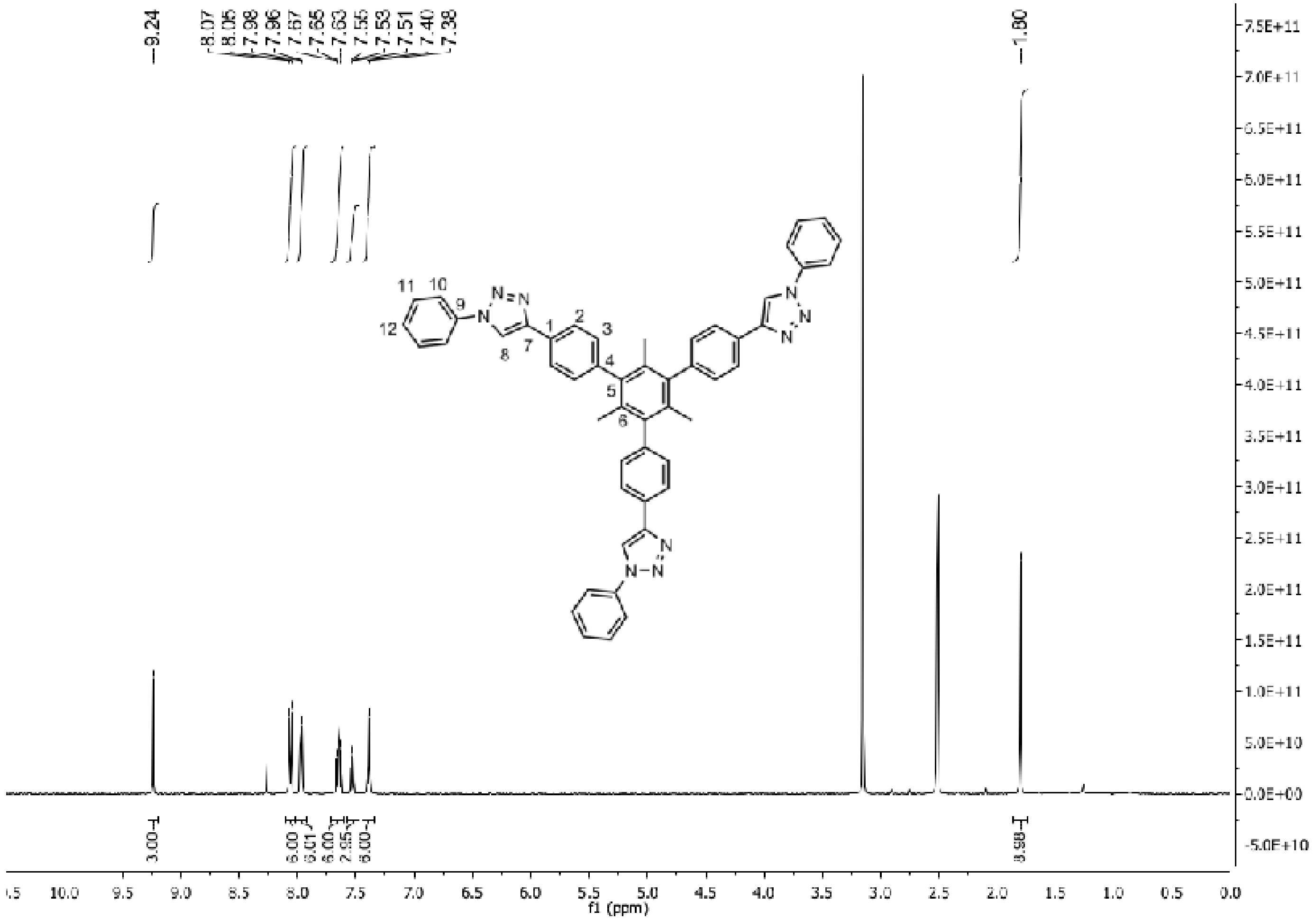

Figure S17 – The ^{1}H NMR spectrum of **11**, measured in DMSO-*d6*.

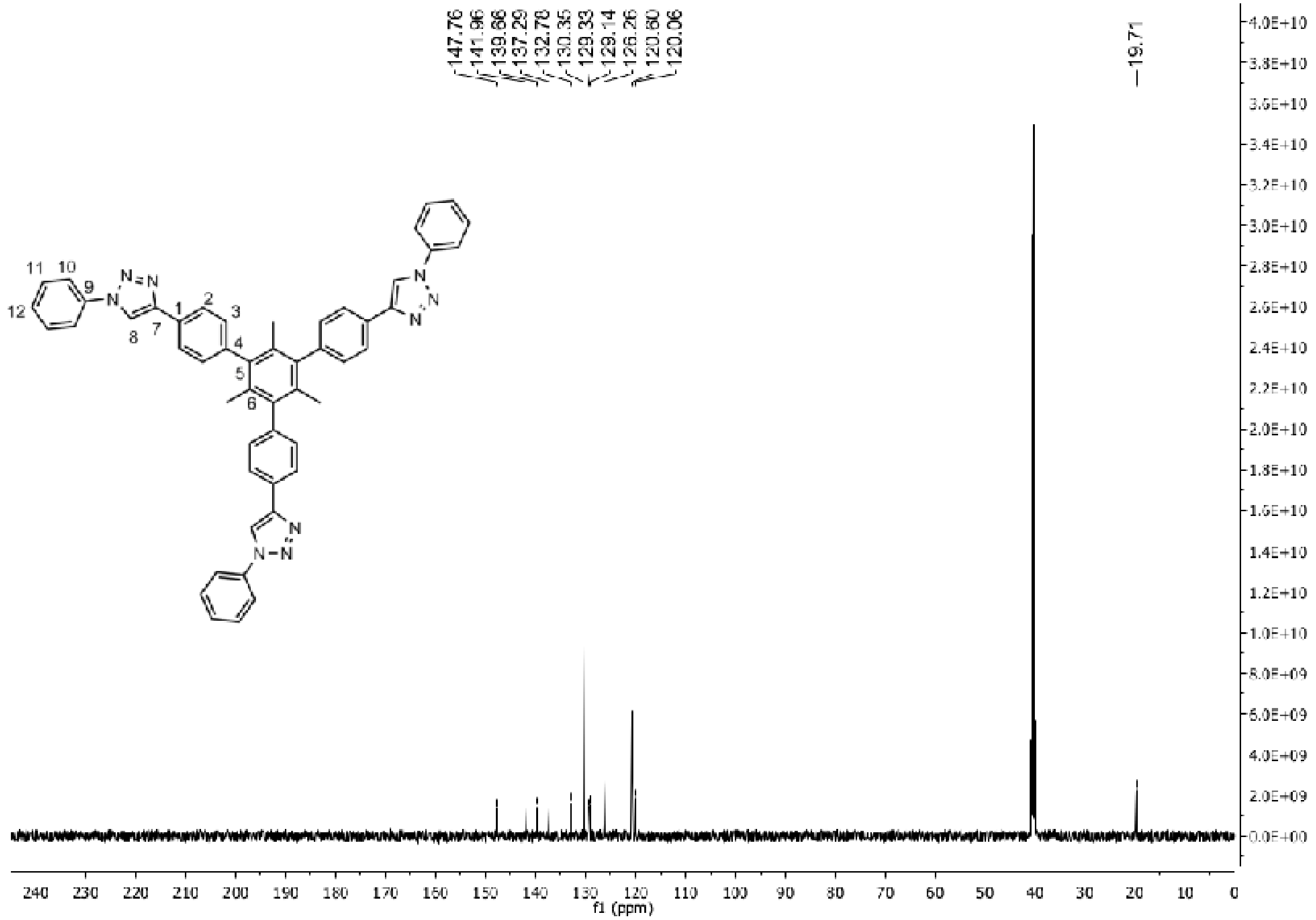

Figure S18 – The ^{13}C NMR spectrum of **11**, measured in DMSO-*d6*.

EI-MS Spectra

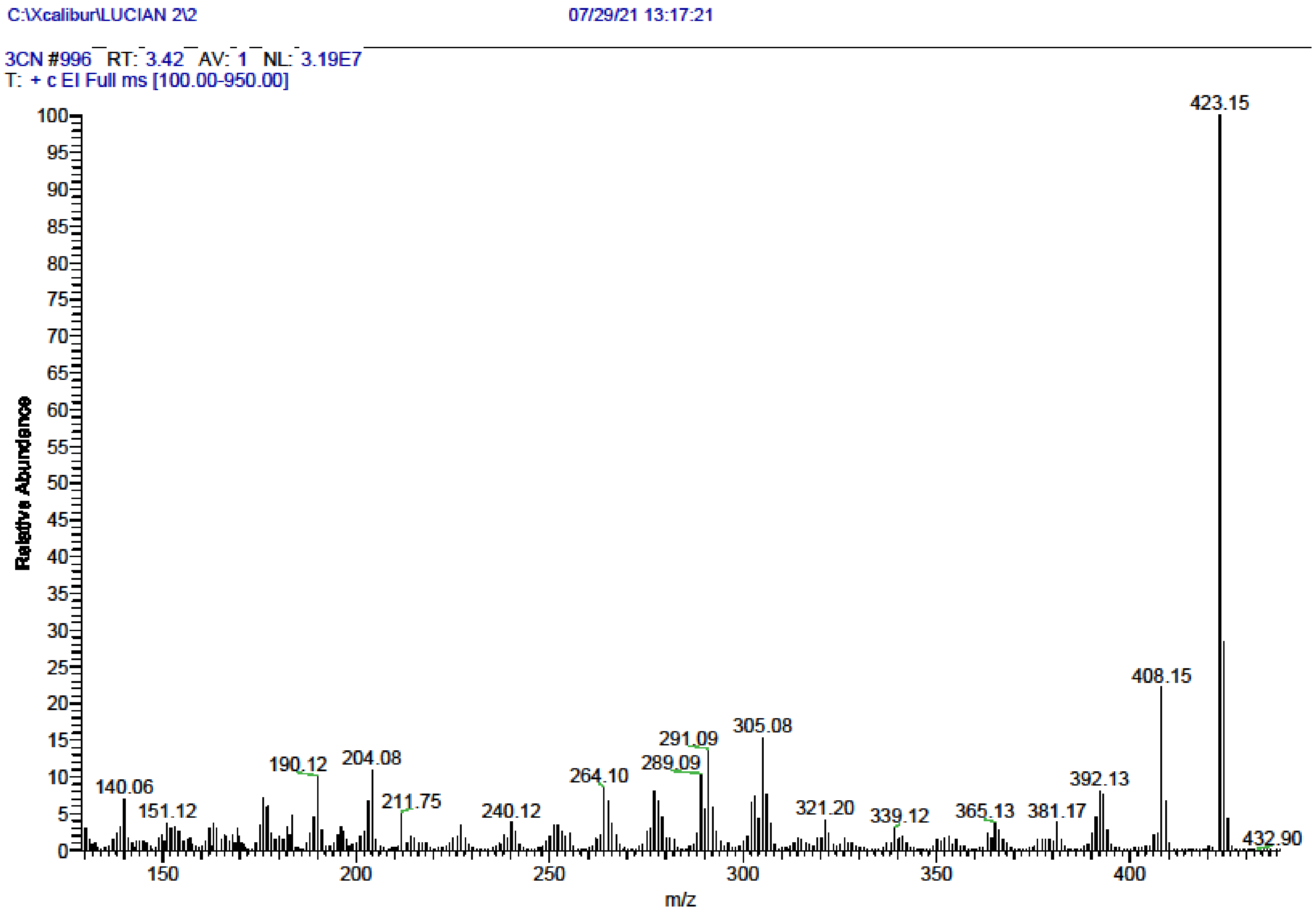

Figure S19 – The EI-MS spectrum of **2**.

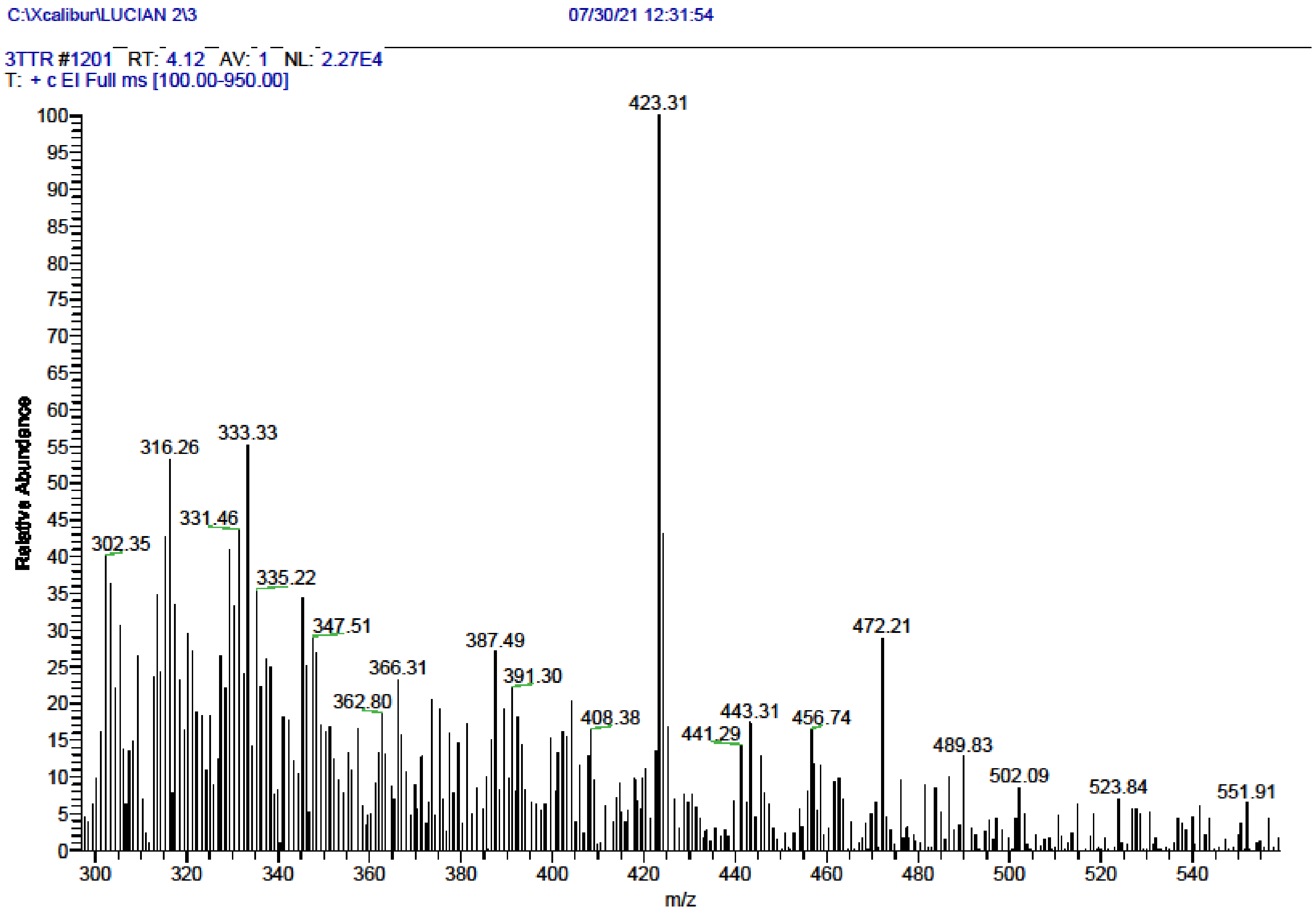

Figure S20 - The EI-MS spectrum of **3**.

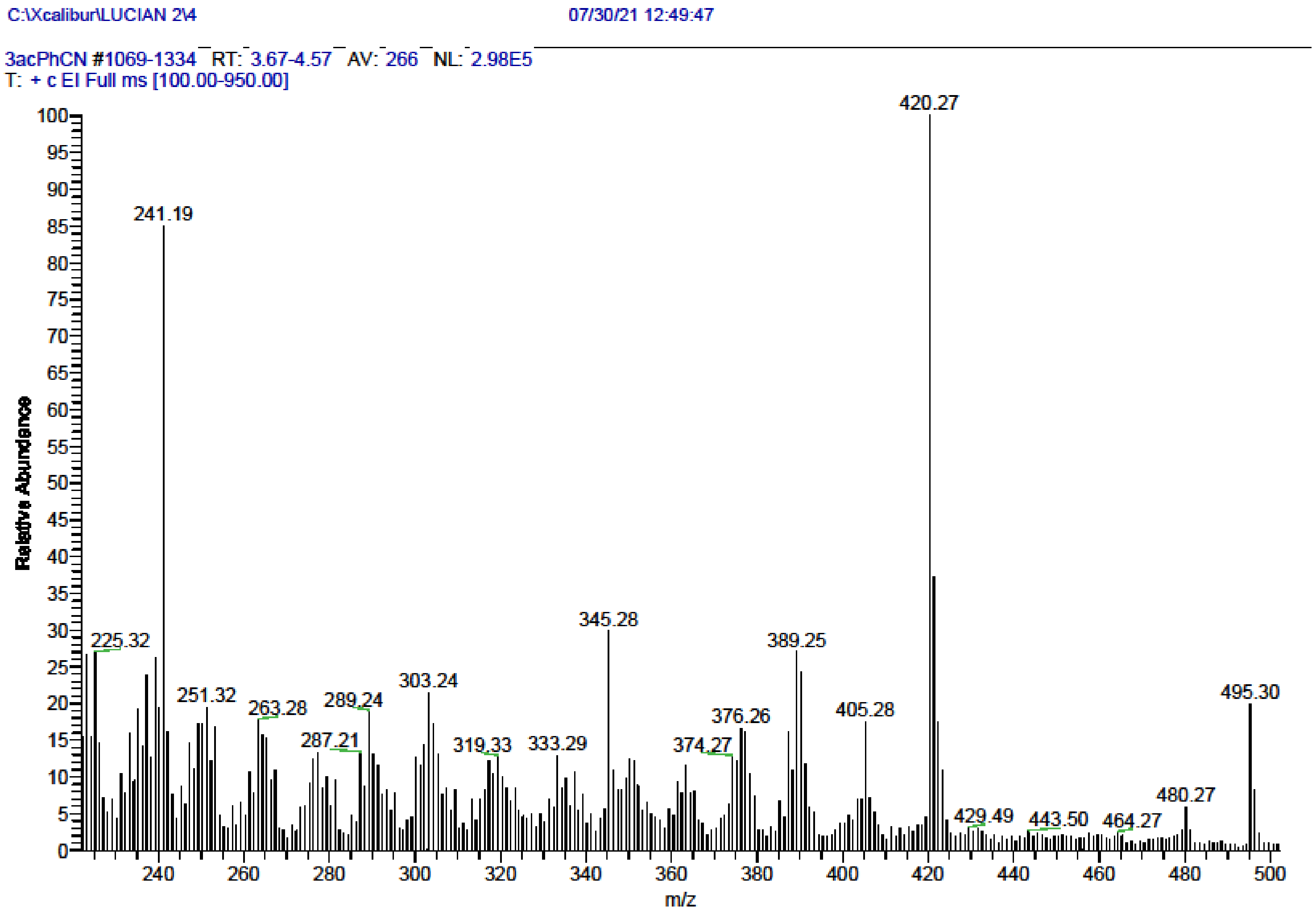

Figure S21 - The EI-MS spectrum of **4**.

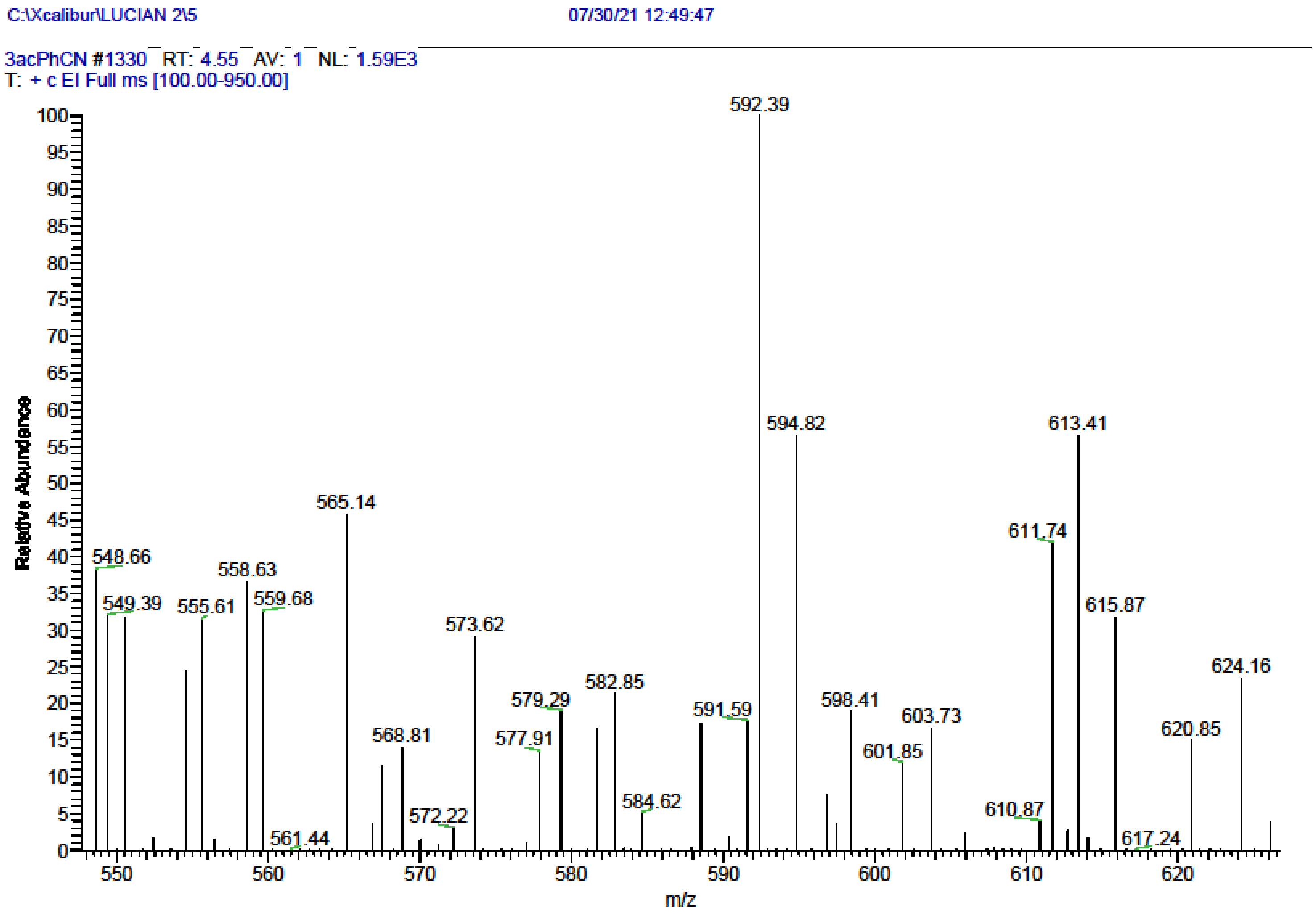

Figure S22 - The EI-MS spectrum of **5**.

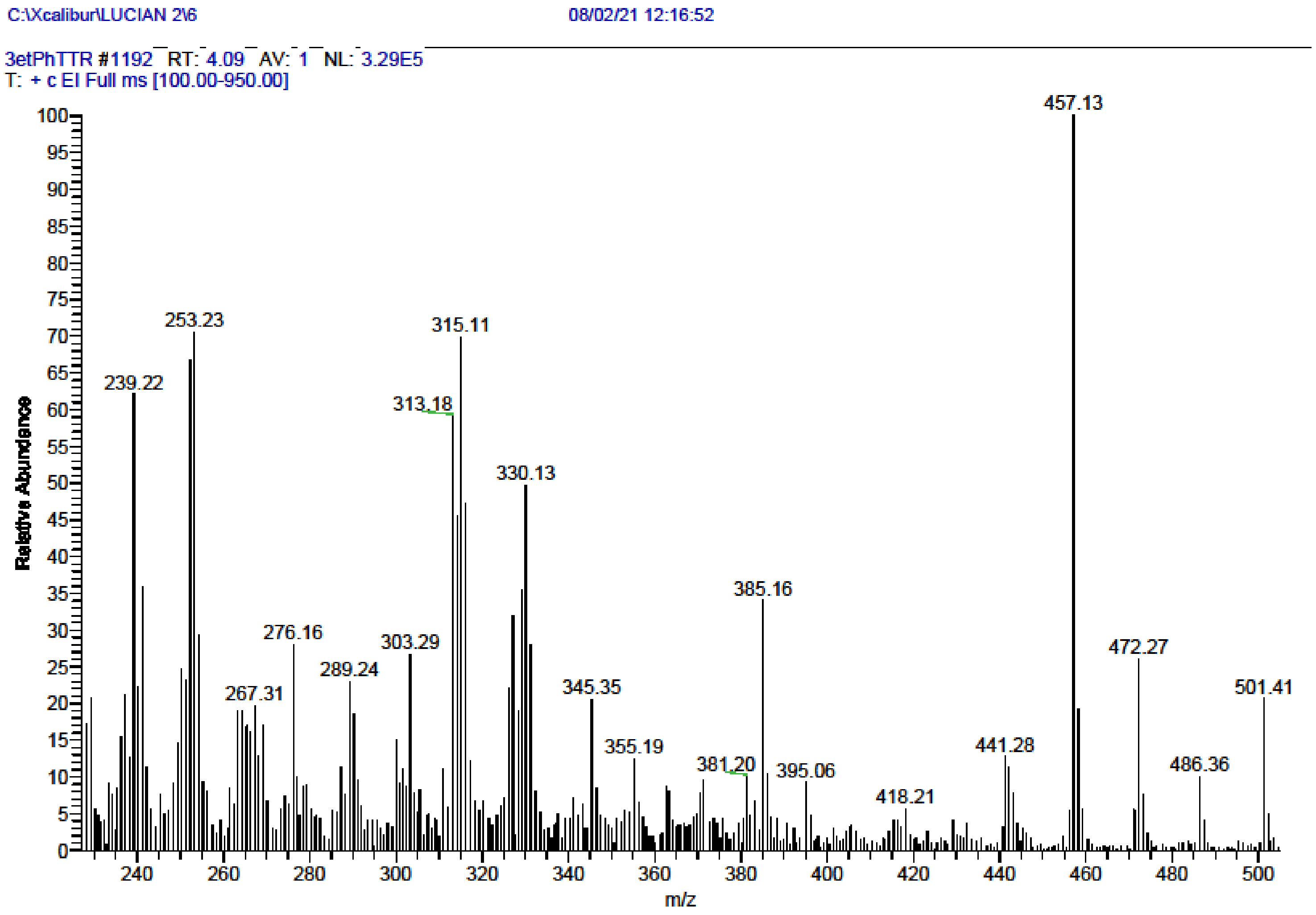

Figure S23 - The EI-MS spectrum of **6**.

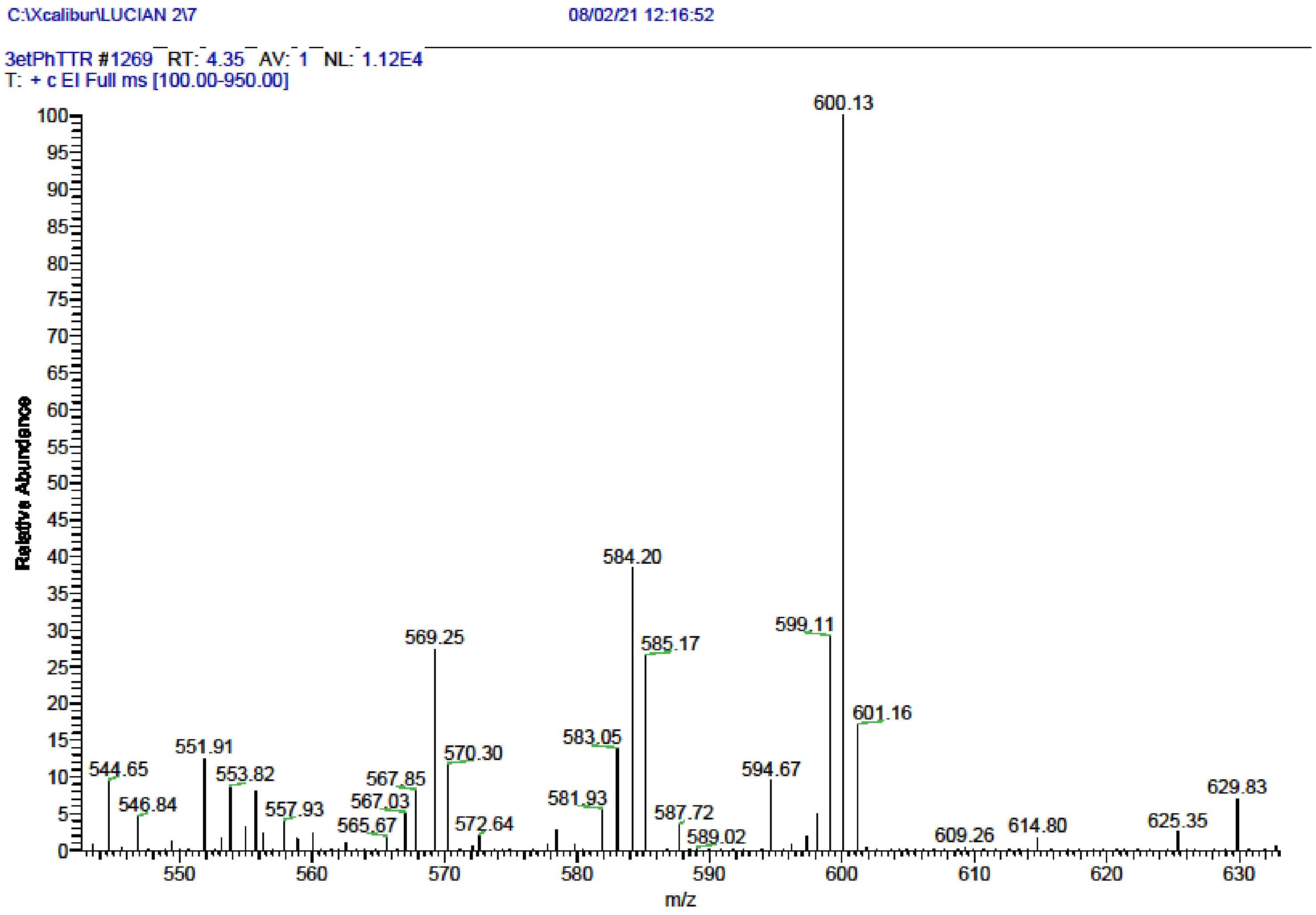

Figure S24 - The EI-MS spectrum of **7**.

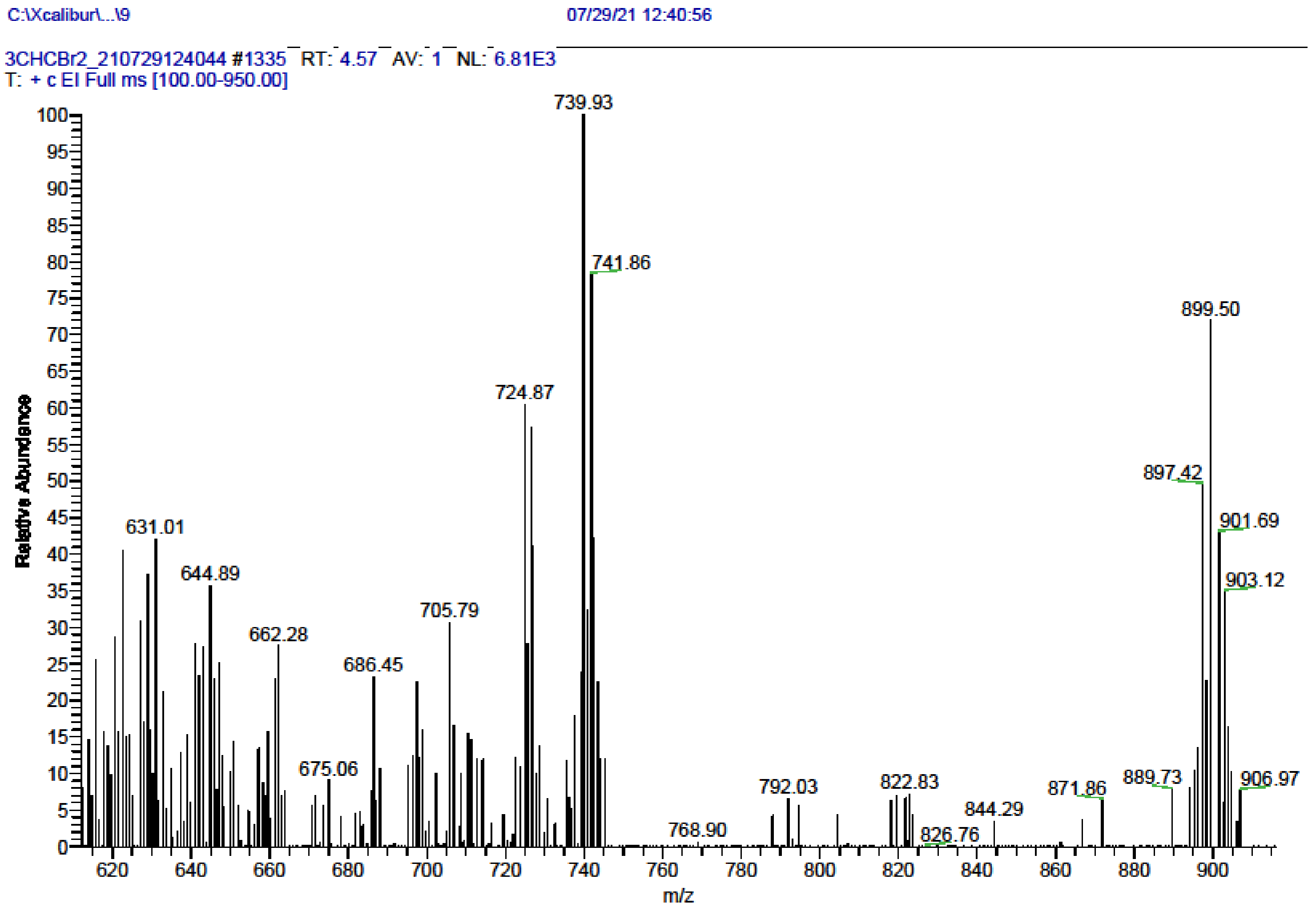

Figure S25 - The EI-MS spectrum of **9**.

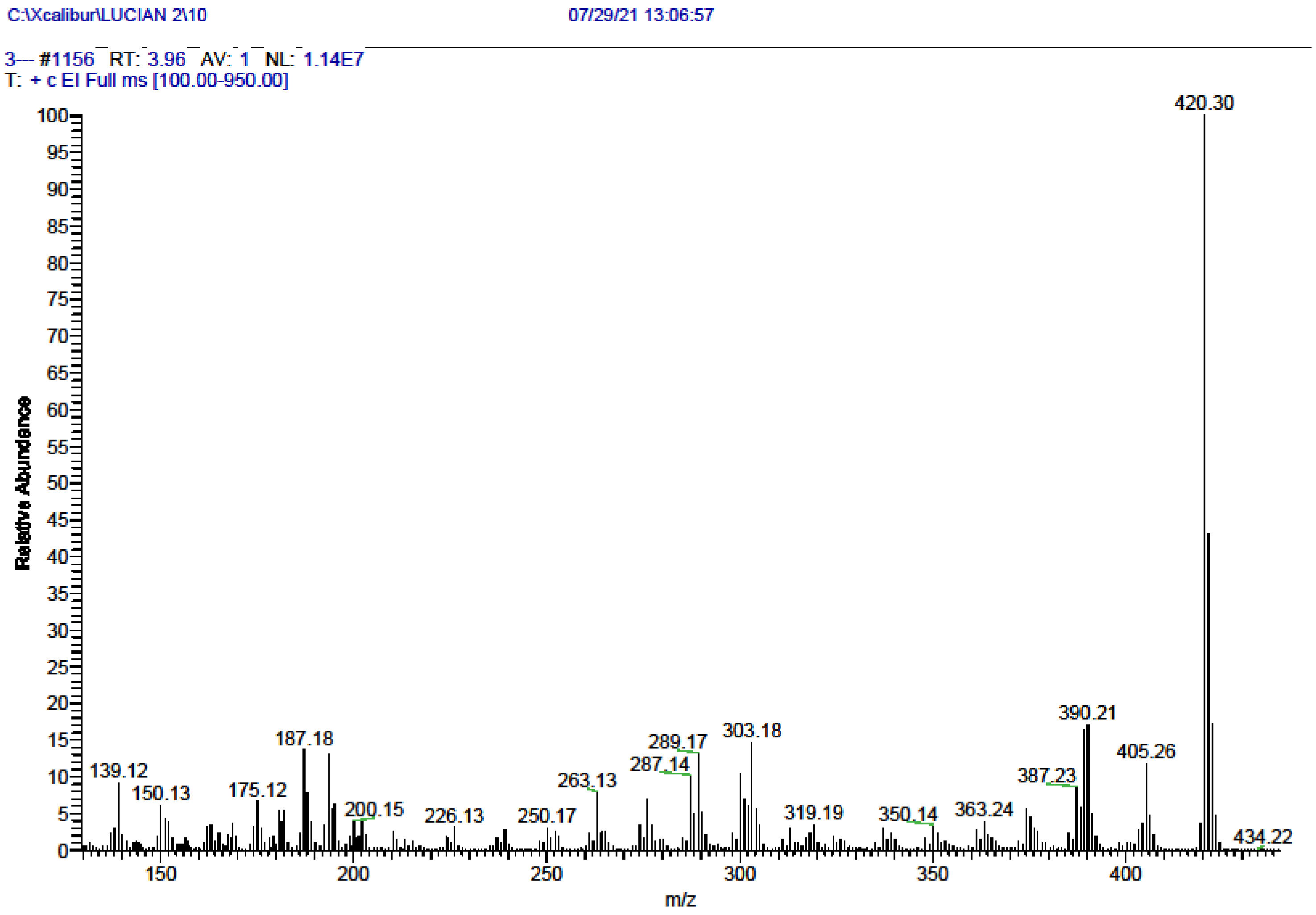

Figure S26 - The EI-MS spectrum of **10**.

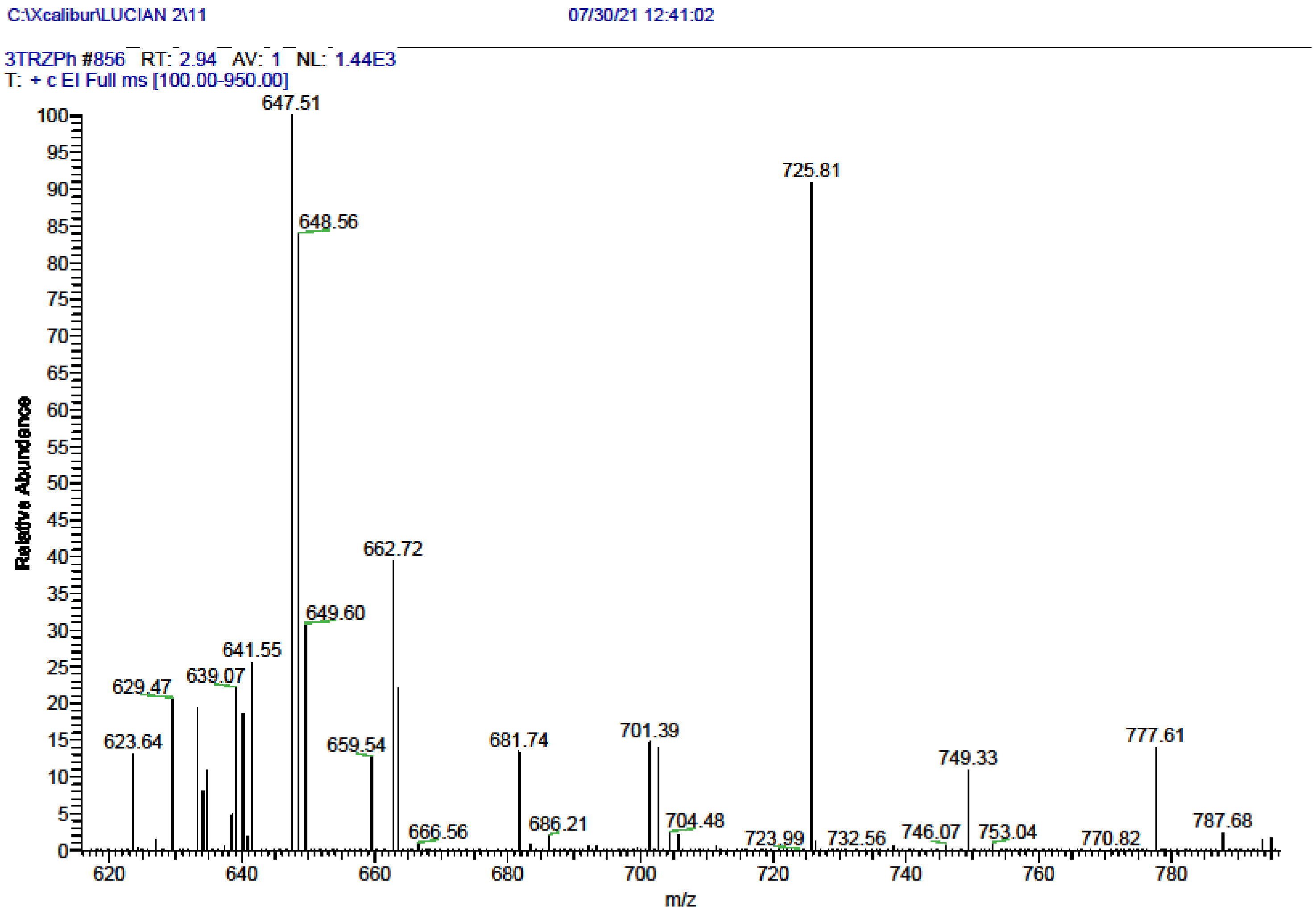

Figure S27 - The EI-MS spectrum of **11**.

X-ray crystallography

Table S1: Crystal data and details of data collection

Parameter	**2**	**9**	**10**	**12**
empirical formula	$C_{30}H_{23.25}N_3O_{1.13}$	$C_{33}H_{26.75}Br_6O_{1.5}$	$C_{33}H_{24}$	$C_{55.5}H_{46}Ag_2F_6N_3O_4$
Fw	443.76	926.75	420.52	1148.69
space group	$P2_1/c$	$P2_1/n$	$P2_1/c$	$C2/c$
a [Å]	12.0041(12)	20.0818(18)	12.0152(19)	28.8519(7)
b [Å]	8.3857(4)	7.8589(6)	8.6661(15)	18.1939(5)
c [Å]	30.145(3)	21.949(2)	24.388(3)	19.6865(7)
β [°]	122.388(13)	97.147(10)	97.910(14)	96.354(5)
V [Å^3]	2562.5(5)	3437.1(5)	2515.3(7)	10270.5(5)
Z	4	4	4	8
r_{calcd} [g·cm^{-3}]	1.150	1.791	1.110	1.486
Crystal size [mm]	0.20 × 0.05 × 0.05	0.35 × 0.18 × 0.04	0.35 × 0.10 × 0.05	0.45 × 0.30 × 0.03
T [K]	180	297	293	180
μ [mm^{-1}]	0.071	7.034	0.063	0.832
2Θ range [°]	3.2 to 50.052	5.51 to 50.054	4.462 to 50.054	3.272 to 57.552
Reflections collected	16471	14360	9944	23210
Independent reflections	4503[R_{int}=0.0450]	6050[R_{int}=0.1057]	4446[R_{int}=0.0564]	11697[R_{int}=0.0356]
Data/restraints/parameter	4503/0/333	6050/0/332	4446/0/301	11697/54/655
R_1[a]	0.0670	0.0870	0.0764	0.0539
wR_2[b]	0.1827	0.2768	0.2225	0.1137
GOF[c]	1.027	0.999	1.004	1.024
Largest diff. peak/hole / e	0.44/-0.24	1.78/-1.28	0.20/-0.15	0.69/-0.84
CCDC No.	2099115	2099116	2099117	2106774

[a] $R_1 = \Sigma||F_o| - |F_c||/\Sigma|F_o|$. [b] $wR_2 = \{\Sigma[w(F_o^2 - F_c^2)^2]/\Sigma[w(F_o^2)^2]\}^{1/2}$. [c] $GOF = \{\Sigma[w(F_o^2 - F_c^2)^2]/(n - p)\}^{1/2}$, where *n* is the number of reflections and *p* is the total number of parameters refined.

Bond distances (Å) and angles(°)

Table S2. Bond distances (Å) for **2**

N1-C1	1.147(4)	C11-C16	1.509(4)
N2-C23	1.144(3)	C12-C13	1.404(3)
N3-C30	1.143(4)	C12-C24	1.498(3)
C1-C2	1.447(4)	C13-C14	1.516(3)
C2-C3	1.384(4)	C17-C18	1.376(4)
C2-C7	1.381(4)	C17-C22	1.377(4)
C3-C4	1.391(4)	C18-C19	1.388(4)
C4-C5	1.394(3)	C19-C20	1.381(4)
C5-C6	1.383(4)	C20-C21	1.365(4)
C5-C8	1.501(3)	C20-C23	1.445(4)
C6-C7	1.379(4)	C21-C22	1.377(4)
C8-C9	1.401(3)	C24-C25	1.390(3)
C8-C13	1.400(3)	C24-C29	1.388(4)
C9-C10	1.402(4)	C25-C26	1.380(4)
C9-C15	1.510(4)	C26-C27	1.381(4)
C10-C11	1.408(3)	C27-C28	1.385(4)
C10-C17	1.502(3)	C27-C30	1.442(4)
C11-C12	1.404(3)	C28-C29	1.381(4)

Table S3. Bond angles(°) for **2**

N1-C1-C2	178.0(3)	C8-C13-C12	119.7(2)
C3-C2-C1	120.4(3)	C8-C13-C14	120.2(2)
C7-C2-C1	119.5(3)	C12-C13-C14	120.1(2)
C7-C2-C3	120.1(2)	C18-C17-C10	122.9(2)
C2-C3-C4	119.7(3)	C18-C17-C22	117.8(2)

C3-C4-C5	120.7(3)	C22-C17-C10	119.3(2)
C4-C5-C8	120.6(2)	C17-C18-C19	120.8(3)
C6-C5-C4	118.1(2)	C20-C19-C18	120.0(3)
C6-C5-C8	121.3(2)	C19-C20-C23	122.0(3)
C7-C6-C5	121.8(2)	C21-C20-C19	119.4(2)
C6-C7-C2	119.6(3)	C21-C20-C23	118.5(2)
C9-C8-C5	120.7(2)	C20-C21-C22	119.9(3)
C13-C8-C5	118.5(2)	C17-C22-C21	121.9(3)
C13-C8-C9	120.8(2)	N2-C23-C20	176.6(3)
C8-C9-C10	118.9(2)	C25-C24-C12	122.0(2)
C8-C9-C15	120.7(2)	C29-C24-C12	120.0(2)
C10-C9-C15	120.4(2)	C29-C24-C25	118.1(2)
C9-C10-C11	121.1(2)	C26-C25-C24	121.4(3)
C9-C10-C17	120.5(2)	C25-C26-C27	119.6(3)
C11-C10-C17	118.2(2)	C26-C27-C28	120.1(2)
C10-C11-C16	120.5(2)	C26-C27-C30	121.0(3)
C12-C11-C10	119.0(2)	C28-C27-C30	118.8(3)
C12-C11-C16	120.6(2)	C29-C28-C27	119.7(3)
C11-C12-C24	120.3(2)	C28-C29-C24	121.1(3)
C13-C12-C11	120.4(2)	N3-C30-C27	178.1(5)
C13-C12-C24	119.3(2)		

Table S4. Bond distances (Å) for **9**

Br1-C1	1.897(10)	C12-C11	1.406(13)
Br2-C1	1.802(13)	C12-C17	1.528(14)
Br3-C25	1.883(12)	C11-C10	1.381(14)
Br4-C25	1.886(13)	C11-C26	1.523(10)
Br5-C33	1.882(13)	C10-C16	1.537(14)
Br6-C33	1.879(10)	C21-C24	1.490(11)

C1-C2	1.305(15)	C21-C20	1.3900
C2-C3	1.489(13)	C21-C22	1.3900
C8-C3	1.3900	C20-C19	1.3900
C8-C7	1.3900	C19-C18	1.3900
C3-C4	1.3900	C18-C23	1.3900
C4-C5	1.3900	C23-C22	1.3900
C5-C6	1.3900	C24-C25	1.307(15)
C6-C7	1.3900	C29-C32	1.460(11)
C6-C9	1.514(10)	C27-C28	1.3900
C9-C14	1.362(13)	C27-C26	1.3900
C9-C10	1.424(14)	C28-C29	1.3900
C14-C13	1.448(14)	C29-C30	1.3900
C14-C15	1.506(14)	C30-C31	1.3900
C13-C12	1.395(14)	C31-C26	1.3900
C13-C18	1.514(11)	C32-C33	1.322(14)

Table S5. Bond angles(°) for **9**

Br2-C1-Br1	111.6(7)	C11-C10-C9	120.5(9)
C2-C1-Br1	120.5(10)	C11-C10-C16	119.5(10)
C2-C1-Br2	127.5(9)	C20-C21-C22	120.0
C1-C2-C3	132.4(11)	C20-C21-C24	115.6(7)
C3-C8-C7	120.0	C22-C21-C24	124.3(7)
C8-C3-C2	124.0(6)	C19-C20-C21	120.0
C4-C3-C2	115.9(6)	C20-C19-C18	120.0
C4-C3-C8	120.0	C19-C18-C13	119.2(6)
C3-C4-C5	120.0	C19-C18-C23	120.0
C4-C5-C6	120.0	C23-C18-C13	120.7(6)
C5-C6-C9	121.9(6)	C18-C23-C22	120.0

C7-C6-C5	120.0	C23-C22-C21	120.0
C7-C6-C9	118.0(6)	C25-C24-C21	133.1(11)
C6-C7-C8	120.0	Br3-C25-Br4	111.9(7)
C14-C9-C6	119.3(8)	C24-C25-Br3	122.0(10)
C14-C9-C10	120.9(9)	C24-C25-Br4	126.1(10)
C10-C9-C6	119.8(8)	C28-C27-C26	120.0
C9-C14-C13	118.7(9)	C29-C28-C27	120.0
C9-C14-C15	122.5(10)	C28-C29-C30	120.0
C13-C14-C15	118.8(9)	C28-C29-C32	115.6(6)
C14-C13-C18	119.0(8)	C30-C29-C32	124.4(6)
C12-C13-C14	120.1(9)	C31-C30-C29	120.0
C12-C13-C18	120.9(9)	C30-C31-C26	120.0
C13-C12-C11	119.9(9)	C27-C26-C11	119.0(6)
C13-C12-C17	119.8(9)	C31-C26-C11	120.5(5)
C11-C12-C17	120.4(9)	C31-C26-C27	120.0
C12-C11-C26	118.8(8)	C33-C32-C29	133.1(10)
C10-C11-C12	119.9(9)	Br6-C33-Br5	112.8(6)
C10-C11-C26	121.3(8)	C32-C33-Br5	124.8(9)
C9-C10-C16	120.0(9)	C32-C33-Br6	122.4(10)

Table S6. Bond distances (Å) for **10**

C1-C2	1.152(5)	C13-C26	1.492(5)
C2-C3	1.446(6)	C14-C15	1.515(5)
C3-C4	1.391(5)	C18-C19	1.369(6)
C3-C8	1.380(6)	C18-C23	1.391(5)
C4-C5	1.379(5)	C19-C20	1.389(6)
C5-C6	1.366(5)	C20-C21	1.379(5)
C6-C7	1.380(5)	C21-C22	1.375(6)
C6-C9	1.501(5)	C21-C24	1.420(6)

C7-C8	1.371(5)	C22-C23	1.383(6)
C9-C10	1.388(5)	C24-C25	1.131(7)
C9-C14	1.400(5)	C26-C27	1.373(5)
C10-C11	1.400(5)	C26-C31	1.383(5)
C10-C16	1.528(5)	C27-C28	1.389(6)
C11-C12	1.408(5)	C28-C29	1.372(6)
C11-C18	1.474(5)	C29-C30	1.372(6)
C12-C13	1.390(5)	C29-C32	1.432(6)
C12-C17	1.515(5)	C30-C31	1.377(5)
C13-C14	1.390(5)	C32-C33	1.145(6)

Table S7. Bond angles(°) for **10**

C1-C2-C3	176.1(6)	C9-C14-C15	120.2(4)
C4-C3-C2	121.0(4)	C13-C14-C9	120.0(4)
C8-C3-C2	120.7(4)	C13-C14-C15	119.8(4)
C8-C3-C4	118.3(4)	C19-C18-C11	121.2(4)
C5-C4-C3	119.1(4)	C19-C18-C23	116.4(5)
C6-C5-C4	122.8(4)	C23-C18-C11	122.3(5)
C5-C6-C7	117.6(4)	C18-C19-C20	122.1(4)
C5-C6-C9	121.5(3)	C21-C20-C19	121.3(5)
C7-C6-C9	120.8(4)	C20-C21-C24	120.6(6)
C8-C7-C6	120.7(4)	C22-C21-C20	116.9(5)
C7-C8-C3	121.4(4)	C22-C21-C24	122.5(5)
C10-C9-C6	119.7(4)	C21-C22-C23	121.8(4)
C10-C9-C14	120.2(4)	C22-C23-C18	121.5(5)
C14-C9-C6	120.1(4)	C25-C24-C21	177.4(8)
C9-C10-C11	120.1(4)	C27-C26-C13	120.2(4)
C9-C10-C16	120.4(4)	C27-C26-C31	117.2(4)
C11-C10-C16	119.5(4)	C31-C26-C13	122.6(4)

C10-C11-C12	119.5(4)	C26-C27-C28	121.9(4)
C10-C11-C18	120.0(4)	C29-C28-C27	120.4(4)
C12-C11-C18	120.5(4)	C28-C29-C32	120.7(5)
C11-C12-C17	119.1(4)	C30-C29-C28	117.9(4)
C13-C12-C11	120.1(4)	C30-C29-C32	121.4(4)
C13-C12-C17	120.8(4)	C29-C30-C31	121.8(4)
C12-C13-C26	119.7(4)	C30-C31-C26	120.8(4)
C14-C13-C12	120.1(4)	C33-C32-C29	177.3(6)
C14-C13-C26	120.1(4)		

Table S8. Bond distances (Å) for **12**

Ag1-O2	2.239(3)	C12-C17	1.401(6)	C41-C43[4]	1.352(7)
Ag1-O4	2.289(4)	C13-C14	1.406(5)	C42-C43	1.328(7)
Ag1-N1	2.338(4)	C13-C20	1.511(6)	C44-C45	1.443(9)
Ag1-N3[1]	2.274(4)	C14-C15	1.398(6)	C45-C46	1.362(9)
Ag2-O1	2.281(3)	C14-C33	1.491(6)	C45-C50	1.388(10)
Ag2-O3	2.219(3)	C15-C16	1.399(6)	C46-C47	1.375(9)
Ag2-N2[2]	2.343(4)	C15-C19	1.514(5)	C47-C48	1.385(11)
F1-C2	1.310(6)	C16-C17	1.405(5)	C48-C49	1.384(13)
F2-C2	1.315(5)	C16-C26	1.500(5)	C49-C50	1.319(10)
F3-C2	1.317(5)	C17-C18	1.509(6)	C51-C52	1.25(2)
F4-C4	1.389(6)	C21-C22	1.367(6)	C52-C53[5]	1.398(8)
F5-C4	1.331(7)	C21-C26	1.366(6)	C52-C53	1.403(9)
F6-C4	1.337(6)	C22-C23	1.369(6)	C53-C54	1.392(9)
O1-C1	1.234(5)	C23-C24	1.364(6)	C53-C56[5]	1.386(8)
O2-C1	1.230(5)	C23-C27	1.437(6)	C54-C55	1.389(9)
O3-C3	1.234(5)	C24-C25	1.386(6)	C55-C56	1.402(10)
O4-C3	1.234(6)	C25-C26	1.373(6)	C57-C58	1.47(3)
N1-C5	1.142(5)	C28-C29	1.374(7)	C58-C59	1.46(4)

N2-C27	1.145(5)	C28-C33	1.385(7)	C58-C60[3]	1.77(4)
N3-C34	1.126(6)	C29-C30	1.367(7)	C58-C62[3]	0.60(3)
C1-C2	1.535(6)	C30-C31	1.373(6)	C58-C63	1.35(3)
C3-C4	1.475(9)	C30-C34	1.450(6)	C59-C60[3]	0.57(4)
C5-C6	1.432(6)	C31-C32	1.377(6)	C59-C60	1.28(4)
C6-C7	1.376(7)	C32-C33	1.369(6)	C59-C61[3]	1.05(4)
C6-C11	1.388(6)	C35-C36[3]	1.3892(10)	C60-C61	1.39(4)
C7-C8	1.386(6)	C35-C36	1.3891(10)	C61-C62	1.40(3)
C8-C9	1.381(6)	C36-C37	1.3892(10)	C62-C63	1.39(3)
C9-C10	1.374(6)	C37-C38	1.3898(10)	C62-C63[3]	1.39(3)
C9-C12	1.495(6)	C38-C39	1.329(18)	C63-C63[3]	0.000(16)
C10-C11	1.388(6)	C40-C41	1.438(9)		
C12-C13	1.389(6)	C41-C42	1.359(7)		

[1]1/2+X,1/2+Y,+Z; [2]1/2-X,-1/2+Y,1/2-Z; [3]1-X,+Y,1/2-Z; [4]1/2-X,3/2-Y,-Z; [5]1/2-X,3/2-Y,1-Z

Table S9. Bond angles(°) for **12**

O2-Ag1-O4	122.83(16)	C13-C14-C33	118.7(4)	C50-C49-C48	121.0(10)
O2-Ag1-N1	116.39(15)	C15-C14-C13	121.0(4)	C49-C50-C45	121.4(8)
O2-Ag1-N3[1]	115.41(14)	C15-C14-C33	120.3(4)	C51-C52-C53[7]	121.8(9)
O4-Ag1-N1	105.20(15)	C14-C15-C16	118.9(4)	C51-C52-C53	125.3(8)
N31-Ag1-O4	96.99(15)	C14-C15-C19	120.7(4)	C537-C52-C53	112.9(14)
N31-Ag1-N1	95.10(15)	C16-C15-C19	120.3(4)	C527-C53-C52	67.1(14)
O1-Ag2-N2[2]	96.30(13)	C15-C16-C17	121.1(4)	C567-C53-C54	171.1(13)
O3-Ag2-O1	147.10(11)	C15-C16-C26	119.7(3)	C55-C54-C53	139.6(16)
O3-Ag2-N2[2]	115.51(13)	C17-C16-C26	119.2(4)	C54-C55-C56	99.4(16)
C1-O1-Ag2	125.4(3)	C12-C17-C16	118.6(4)	C537-C56-C55	128.2(16)
C1-O2-Ag1	124.2(3)	C12-C17-C18	120.3(4)	C57-C58-C60[5]	122(2)
C3-O3-Ag2	121.0(3)	C16-C17-C18	121.0(4)	C59-C58-C57	116(2)
C3-O4-Ag1	117.7(3)	C26-C21-C22	122.3(4)	C59-C58-C60[5]	17.0(14)

C5-N1-Ag1	146.6(4)	C21-C22-C23	119.5(5)	C625-C58-C57	42(5)
C27-N2-Ag2[3]	152.8(4)	C22-C23-C27	120.8(4)	C625-C58-C59	157(6)
C34-N3-Ag1[4]	168.0(4)	C24-C23-C22	119.6(4)	C625-C58-C60[5]	153(6)
O1-C1-C2	116.8(4)	C24-C23-C27	119.6(4)	C625-C58-C63	81(5)
O2-C1-O1	129.9(4)	C23-C24-C25	120.2(4)	C63-C58-C57	122.4(19)
O2-C1-C2	113.2(4)	C26-C25-C24	120.7(5)	C63-C58-C59	122(2)
F1-C2-F2	107.6(4)	C21-C26-C16	122.0(4)	C63-C58-C60[5]	113(2)
F1-C2-F3	106.6(5)	C21-C26-C25	117.7(4)	C60-C59-C58	110(3)
F1-C2-C1	113.6(4)	C25-C26-C16	120.3(4)	C605-C59-C58	114(7)
F2-C2-F3	106.5(4)	N2-C27-C23	178.4(5)	C605-C59-C60	60(7)
F2-C2-C1	111.6(4)	C29-C28-C33	121.0(5)	C605-C59-C61[5]	115(7)
F3-C2-C1	110.5(4)	C30-C29-C28	120.0(5)	C615-C59-C58	47(2)
O3-C3-C4	115.2(5)	C29-C30-C31	120.2(4)	C615-C59-C60	155(4)
O4-C3-O3	130.4(5)	C29-C30-C34	118.9(4)	C59-C60-C61	134(4)
O4-C3-C4	114.4(5)	C31-C30-C34	120.9(4)	C585-C61-C57[5]	66.7(19)
F4-C4-C3	110.2(6)	C30-C31-C32	119.1(4)	C585-C61-C60	91(3)
F5-C4-F4	102.0(6)	C33-C32-C31	122.0(4)	C585-C61-C62	23.7(18)
F5-C4-F6	111.5(8)	C28-C33-C14	120.1(4)	C60-C61-C57[5]	156(2)
F5-C4-C3	112.9(6)	C32-C33-C14	122.1(4)	C60-C61-C62	113(2)
F6-C4-F4	101.3(6)	C32-C33-C28	117.7(4)	C62-C61-C57[5]	44.2(13)
F6-C4-C3	117.1(5)	N3-C34-C30	177.1(5)	C575-C62-C61	73.4(18)
N1-C5-C6	178.2(5)	C36-C35-C36[5]	107.3(12)	C575-C62-C63[5]	166(3)
C7-C6-C5	119.9(4)	C35-C36-C37	129.9(10)	C575-C62-C63	166(3)
C7-C6-C11	120.1(4)	C36-C37-C38	116.0(8)	C635-C62-C61	118(2)
C11-C6-C5	120.0(4)	C375-C38-C37	120.5(10)	C63-C62-C61	118(2)
C6-C7-C8	120.3(5)	C39-C38-C37	119.7(5)	C635-C62-C63	0.0(6)
C9-C8-C7	120.2(5)	C39-C38-C37[5]	119.7(5)	C58-C63-C58[5]	97(2)
C8-C9-C12	119.7(4)	C42-C41-C40	116.3(10)	C58-C63-C62	122.0(16)
C10-C9-C8	119.1(4)	C436-C41-C40	120.2(9)	C585-C63-C62	25.4(11)
C10-C9-C12	121.2(4)	C436-C41-C42	123.4(7)	C58-C63-C62[5]	25.4(11)
C9-C10-C11	121.4(4)	C43-C42-C41	116.8(7)	C585-C63-C62[5]	122.0(16)
C10-C11-C6	118.9(5)	C42-C43-C41[6]	119.8(7)	C625-C63-C62	147(2)
C13-C12-C9	119.5(4)	C46-C45-C44	122.2(8)	C635-C63-C58[5]	0(10)
C13-C12-C17	121.4(4)	C46-C45-C50	118.2(7)	C635-C63-C58	0(10)

C17-C12-C9	119.1(4)	C50-C45-C44	119.6(8)	C635-C63-C62	0(10)
C12-C13-C14	119.0(4)	C45-C46-C47	121.5(7)	C635-C63-C62[5]	0(10)
C12-C13-C20	119.8(4)	C46-C47-C48	118.9(8)		
C14-C13-C20	121.2(4)	C49-C48-C47	119.0(8)		

[1]1/2+X,1/2+Y,+Z; [2]1/2-X,-1/2+Y,1/2-Z; [3]1/2-X,1/2+Y,1/2-Z; [4]-1/2+X,-1/2+Y,+Z; [5]1-X,+Y,1/2-Z; [6]1/2-X,3/2-Y,-Z; [7]1/2-X,3/2-Y,1-Z
